# From Conventional to Sustainable Extraction: Improving Phenolic Species Recovery from *Eucalyptus globulus* Leaves

**DOI:** 10.3390/molecules31111927

**Published:** 2026-06-03

**Authors:** Cristina Ott, Raluca Stan, Mihaela Tociu, Alina Morosan, Brindusa Balanuca

**Affiliations:** Department of Organic Chemistry “C. Nenitescu”, Faculty of Chemical Engineering and Biotechnology, National University of Science and Technology POLITEHNICA Bucharest, 011061 Bucharest, Romania; cristina.ott@upb.ro (C.O.); raluca.stan@upb.ro (R.S.); mihaela.tociu@upb.ro (M.T.); alina.morosana@upb.ro (A.M.)

**Keywords:** *Eucalyptus globulus*, sustainable extraction, ultrasound-assisted extraction, microwave-assisted extraction, total polyphenol content, antioxidant activity, FT-ICR MS detection

## Abstract

This research evaluates the influence of extraction method, solvent, and processing time on the recovery of phenolic compounds from *Eucalyptus globulus* leaves and their corresponding antioxidant capacity, through approaches with reduced energy and chemical consumption. Magnetic stirring (MS), and ultrasound- (US) and microwave-assisted (MW) extraction were applied using water or low-ethanol hydroalcoholic systems. Total phenolic content (TPC; Folin–Ciocalteu method) and antioxidant activity (AA; TEAC assay), were assessed to determine the functional properties of the extracts. FT-ICR MS was used to characterize the phytochemical profile. A hydroalcoholic system combined with non-conventional techniques improved extraction efficiency compared to an aqueous system and reduced the processing time. Among the investigated MW conditions, extraction at 360 W for 30 s provided the highest TPC and AA values (252 mg GAE/g DM; 65.67 mg TE/g DM), while US provided maximum TPC of 191 mg GAE/g DM (30 min extraction). MS showed the lowest performance (77 mg GAE/g DM). Phenolic acids, flavonoids, and ellagitannins were assigned across evaluated samples, indicating that the used conditions do not influence the extracts’ qualitative composition. Overall, MW extraction enabled rapid phenolic species recovery under short processing times and low-ethanol conditions, representing a promising approach among the investigated extraction systems.

## 1. Introduction

The growing demand for bioactive compounds of natural origin has driven extensive research across pharmaceutical, food, and cosmetic sectors. This trend is closely associated with the development of sustainable processes, aiming to reduce the energy and solvent consumption, minimize waste generation and enable the production of high value-added products.

In this context, plant-derived materials have attracted considerable attention as sources of biologically active compounds. Among them, *Eucalyptus globulus* leaves represent a rich source of bioactive constituents, including both volatile and non-volatile compounds. Eucalyptus species have been extensively investigated for their essential oil content, particularly due to their antimicrobial and aromatic properties, with numerous studies focusing on their extraction and applications [1,2,3,4,5]. Beyond volatile compounds, eucalyptus leaves also contain significant amounts of phenolic compounds, including flavonoids, phenolic acids, and tannins, which are widely recognized for their functional properties, especially their antioxidant capacity. Compared to essential oils, these non-volatile constituents have received relatively less attention in terms of efficient and sustainable extraction. In recent years, *Eucalyptus globulus* extracts have been investigated or explored in different product formulations as functional ingredients [6,7,8,9,10,11,12]. However, the sustainable recovery of these bioactive molecules requires optimized extraction strategies that ensure both high efficiency and preservation of the phytochemical profile.

Conventional extraction methods applied for eucalyptus leaves and also for other plant materials, often associated with high solvent consumption and long processing times, may have a negative environmental impact [13,14,15]. As a result, increasing attention has been directed toward alternative extraction techniques that align either to green chemistry or sustainability principles [16,17].

In recent years, leaves of different eucalyptus species have been studied due to their complex phytochemical profile and significant antioxidant potential [12,18,19,20,21,22,23]. Various extraction techniques have been applied, including conventional solvent extraction, ultrasound-assisted methods, and accelerated solvent extraction. However, most of these approaches are still based on relatively long extraction times, considerable solvent consumption, and, in some cases, elevated temperature or pressure conditions. Consequently, although efficient in terms of yield, these methods do not fully address the current requirements for sustainable processing.

Thus, the development of extraction strategies based on reduced processing time, lower solvent usage, and improved energy efficiency remains insufficiently explored. Modern extraction techniques, such as microwave-assisted and ultrasound-assisted extraction, have emerged as promising alternatives, offering reduced extraction time, enhanced mass transfer, and improved energy efficiency. In addition, the use of lower amounts of organic solvents contributes to decreasing the environmental footprint of the extraction process, while preserving the integrity of sensitive phytochemicals. Compared to conventional approaches, these techniques enable a more sustainable recovery of phenolic compounds from plant matrices.

In the present study, particular emphasis was placed on the investigation of extraction conditions involving reduced solvent usage, short processing times, and the application of non-conventional extraction techniques. The influences of extraction method (ultrasound- (US) and microwave-assisted (MW) techniques) and processing time (up to 30 min for US and 30 s for MW) on the recovery of phenolic compounds from *Eucalyptus globulus* leaves were evaluated using water and hydroalcoholic mixtures with reduced ethanol content. The extracts were evaluated for total phenolic content (TPC) and antioxidant activity (AA), while FT-ICR mass spectrometry (FT-ICR MS) was used to tentatively characterize the phytochemical profile.

## 2. Results and Discussions

One aspect to be considered in the present study is the use of commercially available dried *Eucalyptus globulus* leaves intended for infusion preparation. Compared to freshly harvested plant material frequently used in experimental studies, such raw material may present inherent variability due to harvesting conditions, drying procedures, storage, and pre-processing steps, which are not fully controlled and may influence extract composition and reproducibility. However, the use of commercially available plant material may also increase the practical relevance of the study, particularly in countries where fresh eucalyptus leaves are not readily accessible and dried commercial products represent a feasible and accessible raw material source for potential phytochemical, cosmetic, or nutraceutical applications, as previously explored and discussed in the literature [24,25,26,27].

In the context of exploring extraction conditions involving reduced solvent usage and shorter processing times, this study aimed to obtain eucalyptus polyphenolic extracts, either aqueous or hydroalcoholic. For this, unconventional extraction methods, shorter extraction times and reduced amounts of ethanol were employed. The efficiency of the experimental conditions was assessed by TPC and AA determination, establishing the optimal extraction conditions among those involved. In addition, phytochemical analyses were performed on a selection of extract samples.

### 2.1. Dry Matter (DM) Content

**Figure 1:**
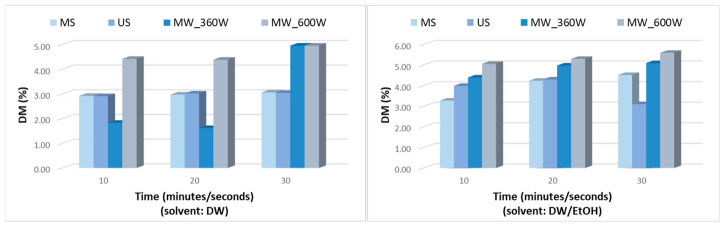
Dry matter content of the aqueous (**left**) and hydroalcoholic (**right**) eucalyptus extracts obtained by different methods. (MS = magnetic stirring; US = ultrasound; MW = microwave).

Figure 1 highlights the influence of the extraction parameters (method, processing time, solvent type) on the generated DM content. The highest DM content (5.59%) was recorded for MW at 600 W, for the longer processing time (30 s), using the hydroalcoholic system. When water was used, the highest DM amount was obtained also for MW (4.96%), irrespective of the power set for the process (360 W or 600 W).

Comparing the methods used, US had an average yield, with comparable DM content for all processing times, while MS led to the lowest amounts of DM extract. The results suggest that in the absence of ethanol, a high energy input—such as that generated by microwaves—becomes essential to favor the release of water-soluble phenolic compounds.

### 2.2. Evaluation of Total Phenolic Content (TPC)

**Figure 2:**
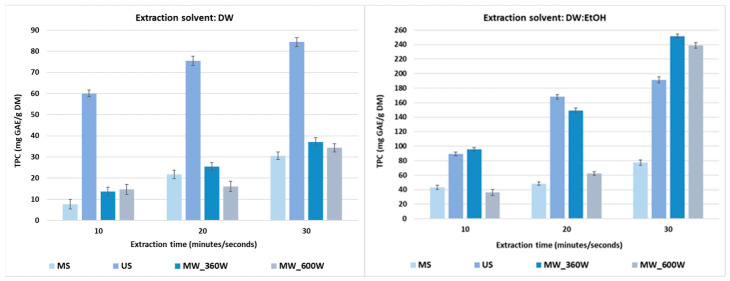
Total phenolic content of aqueous (**left**) and hydroalcoholic (**right**) eucalyptus extracts obtained by different methods. (MS = magnetic stirring; US = ultrasound; MW = microwave).

Determination of TPC in *Eucalyptus globulus* leaf extracts was performed using the Folin–Ciocalteu method [28,29,30], a classic technique in phytochemical evaluation due to its simplicity and sensitivity. The obtained results are shown in Figure 2.

Influence of the extraction method. The extraction method shows an important impact upon the extraction of phenolic compounds (Figure 2). MW extraction provided the highest TPC values among the investigated extraction methods, particularly at 360 W, where the highest TPC value (approximately 252 mg GAE/g DM) was obtained for the hydroalcoholic extract. A comparable value (approx. 239 mg GAE/g DM) was recorded at 600 W, supporting the ability of microwave irradiation to facilitate plant structure disrupting and release of phenolic compounds. US resulted in intermediate TPC values, with the highest value for the hydroalcoholic extract resulting after 30 min of the process (approx. 191 mg GAE/g DM). This result suggests that ultrasounds promote a gradual release of phenolic compounds through cavitation-induced cell disruption. In contrast, MS led to considerably lower TPC values, with a maximum of approx. 77 mg GAE/g DM (DW/EtOH, 30 min), indicating limited extraction efficiency under conventional conditions.

The higher extraction efficiency, correlated with higher values of TPC observed for the extracts obtained by MW and US, may be associated with the specific physicochemical mechanisms characteristic of these techniques, as widely discussed in the literature. Briefly, for the extraction involving microwave, dielectric effects may facilitate a rapid internal heating of the plant matrix and a localized pressure increase within plant cells, facilitating cell wall disruption and the release of intracellular phenolic compounds. In addition, the rapid energy transfer may improve solvent penetration and accelerate the diffusion of target compounds into the extraction medium [31,32]. Ultrasonic extraction is generally related to acoustic cavitation phenomena, which generate microbubbles in the solvent system. The resulting microjets and localized shear forces may enhance mass transfer, increase solvent accessibility to the plant matrix, and facilitate the release of bioactive constituents [33,34,35]. Such mechanisms could contribute to the improved recovery of phenolic compounds, generally observed for MW and US extraction conditions compared to conventional MS extraction.

**Figure 3:**
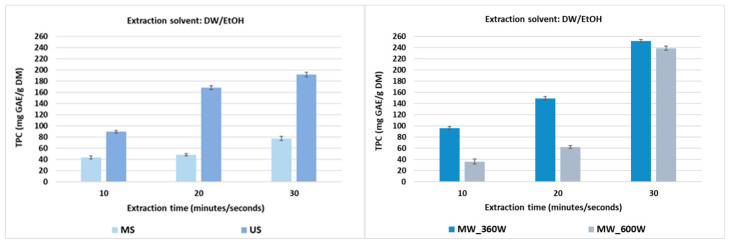
Total phenolic content of hydroalcoholic eucalyptus extracts obtained by different extraction methods. (MS = magnetic stirring; US = ultrasound; MW = microwave).

Influence of the extraction medium. A strong influence of the extraction solvent was observed. A hydroalcoholic system consistently led to higher TPC values compared to water, regardless of the extraction method (Figure 2). While aqueous extraction yielded up to approx. 84 mg GAE/g DM (US, 30 min), the use of a water–ethanol mixture resulted in increased values, reaching up to 250 mg GAE/g DM (MW, 360 W). To better highlight the influence of the extraction medium under experimental conditions, a focused analysis of hydroalcoholic extracts is presented in Figure 3. This graphical representation allows a clearer comparison between extraction methods and processing conditions in terms of phenolic compound recovery.

This behavior may be explained by the intermediate polarity of ethanol–water mixtures, which can facilitate the efficient extraction of both polar and moderately polar phenolic compounds. Compared to water alone, the presence of ethanol may modify the polarity and dielectric properties of the extraction medium, improving the solubilization of moderately polar phenolic compounds [31,36,37]. In MW, ethanol–water mixtures may also influence microwave energy absorption and heat distribution within the extraction system, contributing to improved extraction efficiency under certain conditions [31]. In addition, ethanol may enhance the permeability of plant cell walls and promote the release of intracellular bioactive compounds. Besides reducing solvent consumption, the choice of a low ethanol content (DW/EtOH, 80/20 *v*/*v*) was also intended to maintain relatively mild extraction conditions while improving the recovery of phenolic derivatives under the investigated experimental conditions.

Influence of the extraction time. Extraction time also played an important role upon the phenolic content. For US and MS, an increase in extraction time led to a progressive increase in TPC values, suggesting a diffusion-controlled extraction process. In contrast, MW achieved high TPC values within very short processing times (10–30 s), indicating a rapid and efficient release of phenolic compounds. Overall, MW extraction at 360 W for 30 s combined greater TPC values with a short extraction time, representing a promising extraction setup among the investigated systems.

Comparing the TPC of the current eucalyptus extracts with those reported in the literature, we assume that the maximum phenolic content of approx. 252 mg GAE/g DM is lower than other reported experimental data [12,38,39]. Notwithstanding, considering the involved experimental parameters, e.g., low ethanol content in water for the hydroalcoholic system and low extraction time, the current optimized experimental conditions may represent milder extraction approaches compared to studies involving higher ethanol content (50% [12]/30–70% [40]), longer processing times of up to 2 h ([12,38,40]) or increased temperature and pH adjustment ([39,40]). In other words, a lower TPC value associated with reduced phenolic species recovery from the plant source may represent an acceptable compromise when considering extraction conditions involving shorter processing times, reduced ethanol proportions, and a relatively lower estimated energy requirement.

### 2.3. Relationship Between Recovered Dry Matter and Phenolic Content

The observations related to TPC are further supported by the calculation of the TPC/DM ratio, here proposed as an exploratory empirical parameter intended to comparatively assess the relationship between phenolic content and dry matter recovery under the investigated extraction conditions. This index is not a direct measure of extraction selectivity, but rather an indicative comparative tool reflecting the relative enrichment of phenolic content within the recovered dry matter. Even if it is not always explicitly expressed as a ratio, similar concepts of phenolic yield or extraction efficiency have been discussed in the literature [36,37].

**Figure 4:**
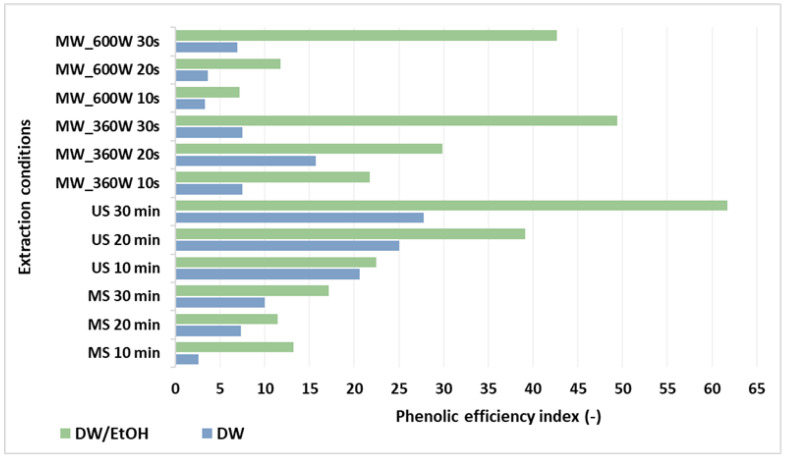
Phenolic efficiency index (expressed as TPC/DM) of eucalyptus extracts obtained under different extraction methods and processing conditions. (MS = magnetic stirring; US = ultrasound; MW = microwave).

Generally, the results revealed an increase in TPC/DM values with increasing extraction time for all tested methods when DW/EtOH was used (Figure 4). Since DM was determined once for each whole lyophilized extract, error bars were not included for the TPC/DM ratio.

Comparing the investigated extraction techniques, US showed the highest TPC/DM values, reaching a maximum value of 61.70 for the longest extraction time (30 min). This suggests that prolonged ultrasound treatment increased the proportion of Folin–Ciocalteu-reactive compounds within the recovered dry matter under the investigated conditions. In contrast, MW extraction enabled rapid recovery of phenolic compounds, particularly at 360 W, where a TPC/DM value of approximately 49 was obtained after only 30 s of extraction. Increasing microwave power to 600 W did not result in a proportional increase in TPC/DM values, suggesting that moderate energy input may provide favorable conditions for rapid phenolic species recovery. In contrast, conventional MS extraction conduces to considerably lower TPC/DM values (11–17), indicating lower extraction efficiency of phenolic derivatives.

Overall, the TPC/DM ratio indicated differences according to the extraction method and processing conditions. Although this empirical parameter should not be interpreted as a direct measure of extraction selectivity, the results suggest that MW favored a more rapid recovery of phenol species, whereas US extraction under longer processing times resulted in comparatively higher TPC/DM values. Moreover, the higher TPC/DM values observed for hydroalcoholic extracts compared with aqueous extracts further highlight the influence of solvent composition on phenolic compounds’ recovery behavior.

### 2.4. Assessment of Antioxidant Activity (AA)

The antioxidant activity (AA) of the eucalyptus extracts was evaluated to assess the functional relevance of the obtained extracts. Given the known antioxidant potential of phenolic compounds, a correlation between TPC and AA values was expected. In general, higher TPC values were associated with increased AA, suggesting that phenolic compounds may represent important contributors to the radical scavenging capacity of the extracts.

**Figure 5:**
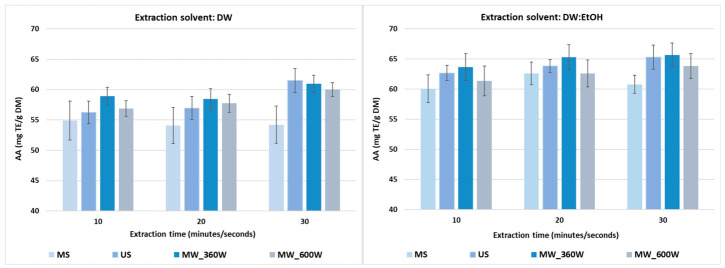
Total antioxidant activity of aqueous (**left**) and hydroalcoholic (**right**) eucalyptus extracts obtained by different extraction methods. (MS = magnetic stirring; US = ultrasound; MW = microwave).

As shown in Figure 5, the determined AA values were generally higher for hydroalcoholic extracts compared to aqueous ones, suggesting differences in the recovery of compounds contributing to antioxidant activity under the investigated extraction conditions. MW at 360 W resulted in the highest AA (up to 65.67 mg TE/g DM), in agreement with the elevated phenolic content. However, increasing the microwave power to 600 W did not lead to a proportional increase in antioxidant activity, which may suggest partial alteration or reduced contribution of some antioxidant species under higher energy input conditions. US showed a gradual increase in AA values with extraction time, following a similar trend to that observed for TPC and the TPC/DM ratio. In the case of MS, lower AA values were generally observed, while prolonged processing times may have influenced the stability or recovery of some antioxidant compounds.

Some fluctuations in AA values may suggest a complex relationship between extraction efficiency and compound stability, rather than a strictly linear dependence on TPC. The weaker proportionality observed for some extraction conditions may indicate that the qualitative composition and antioxidant efficiency of the recovered compounds can be partially affected by prolonged extraction. This non-proportional behavior was particularly evident for US extracts, where high TPC values were not accompanied by equivalent increases in AA. Similar observations have been reported for ethanolic ultrasound-assisted extraction systems, where some individual phenolic compounds from *Eucalyptus globulus* decreased at longer extraction times compared with shorter treatments [24]. Therefore, antioxidant behavior may depend not only on total Folin–Ciocalteu-reactive phenolic content. A Pearson correlation analysis performed by using the experimental TPC and AA values indicated a moderate positive correlation between the two parameters (Pearson’s r = 0.580), suggesting that the relative abundance of individual phenolic classes and their distinct redox capacities may additionally influence antioxidant behavior.

### 2.5. Phytochemical Profile

The phytochemical composition of *Eucalyptus globulus* extracts was investigated by means of FT-ICR MS to trace the profile of bioactive compounds who may support the trends observed for TPC and AA. For this analysis, five eucalyptus extracts were selected, based on their antioxidant performance. The analyzed samples and their corresponding acronyms are presented in Table 1.

The FT-ICR MS analysis suggested a complex phytochemical profile, with well-recognized compounds with biological activity, such as phenolic acids, flavonoids, and hydrolysable tannins, detected in all analyzed samples (E1–E5), consistent with the literature regarding the phytochemical composition of *Eucalyptus globulus* leaves, particularly in ethanolic and aqueous extracts [12,41]. The tentatively assigned compounds, listed in Table 2, were detected in both positive and negative electrospray ionization modes (ESI+ and ESI−), depending on their chemical structure and ionization behavior. Electrospray ionization enables the formation of ions through protonation or deprotonation processes occurring in solution and during the transfer to the gas phase [42]. In this context, phenolic compounds may be detected in either ionization mode depending on their structural features and functional groups. Due to their acidic character, phenolic acids are often reported to be preferentially detected in ESI− mode, while flavonoids or glycosylated derivatives tend to form protonated or adduct ions in ESI+ mode [43,44].

The present dataset, however, indicates a more complex ionization behavior. Several compounds, including ellagic acid, quercetin derivative, and hydrolysable tannins (e.g., corilagin, pedunculagin), were detected in both ionization modes, whereas others were observed mainly in ESI+ mode. This distribution suggests that ionization efficiency is influenced not only by compound class, but also by specific structural features, including the presence of multiple functional groups and conjugation patterns.

In the case of assigned hydrolysable tannins, some compounds were detected in both ESI− and ESI+ modes, while others were observed only in ESI+ mode. This behavior may be related to structural differences between these compounds. For instance, dimethylellagic glucoside contains methyl substituents that may reduce the ability of the molecule to undergo deprotonation. In contrast, compounds, such as corilagin, pedunculagin and tellimagrandin I, possess multiple free phenolic hydroxyl groups, which may favor the formation of stable deprotonated ions in ESI− mode. In addition, ion suppression effects in complex mixtures may further influence the detection of certain compounds, particularly in negative ionization mode, where competition for charge can occur [47]. Detected flavonoids (e.g., quercetin, quercitrin, catechin/epicatechin) and structurally complex phenolic derivatives (unassigned) were found mainly in ESI+ mode, reflecting their tendency to form protonated or adduct ions under the applied FT-ICR MS conditions.

To further support the qualitative FT-ICR MS assignments, the structures of representative compounds of different chemical classes are presented in Figure 6, and their corresponding mass spectra in Figure 7, while the complete set of spectra is provided in the Supplementary Material (Figures S1–S31).

Some of the molecular formulas could not be assigned unambiguously based only on accurate mass measurements. For these compounds, tentative classification was performed by considering molecular formulas and comparison with data in the literature. For instance, highly oxidized polyphenolic structure C_21_H_10_O_13_ was associated with hydrolysable tannins, as similar valoneic acid dilactone have been previously reported in *Eucalyptus globulus* leaves [45], while molecular formula C_14_H_10_O_10_, also a highly oxidized polyphenolic structure, is potentially related to a digalloyl moiety or a dimer of gallic acid, which is identified as a key building block and precursor for hydrolysable tannins found in *Eucalyptus globulus* [46].

FT-ICR MS analysis provided qualitative compositional information and did not allow direct quantitative comparison of compound abundance between samples under the present experimental conditions. Therefore, similar spectral features observed for the investigated extracts (E1–E5) should not be interpreted as evidence of identical concentrations of the tentatively assigned compounds, since ionization efficiency and signal response may influence the relative spectral intensities. The presence of similar peaks in all registered spectra suggests that, in general, the phytochemical composition of *Eucalyptus globulus* leaves was preserved regardless of the extraction method and extraction medium, and the differences observed in the TPC and AA tests were more likely related to the extraction efficiency and relative distribution of the compounds.

### 2.6. Estimated Specific Energy Demand Related to Phenolic Species Recovery

It should be noted that the sustainability of an extraction process is a multifactorial concept that cannot be fully assessed solely based on extraction methods, time or solvent composition. Nevertheless, shorter extraction times together with reduced ethanol proportion may represent preliminary indicators of improved process efficiency. Additionally, MW and US techniques are frequently discussed in the literature as approaches aligned with green extraction principles, due to their potential to reduce extraction time, solvent consumption, and process intensity under optimized conditions [31,33,34].

A simplified estimated specific energy demand was also calculated [48] by relating the estimated operational energy demand to the amount of recovered phenolic content, using TPC values and dry matter obtained for each extract. Among the investigated extraction methods and using the nominal equipment power, MW extraction showed the lowest estimated SEC_est_ values (Figure S32), reflecting the reduced operational energy demand associated with rapid extraction conditions. Of all the experimental parameters, the lowest SEC_est_ value was obtained for MW extraction at 360 W for 30 s (0.18 kWh/g GAE), whereas the highest values were observed for conventional MS extraction under prolonged processing times (1.44 kWh/g GAE, 30 min). US extraction showed intermediate SEC_est_ values, suggesting a compromise between phenolic compounds recovery and energy demand. These simplified estimations represent comparative exploratory indicators under the investigated laboratory conditions, since no direct experimental measurement of energy consumption or complete sustainability assessment was performed.

## 3. Materials and Methods

### 3.1. Materials

Polyphenolic extracts were obtained from dried eucalyptus leaves (*Eucalyptus globulus* species), which were marketed by Fares Orăştie România (*FARES Bio Vital Laboratories*) as human-use food supplement products (infusion).

As extraction media, distilled water (DW) and, respectively, 80/20 (*v*/*v*) mixture of distilled water and ethanol (96% purity, Sigma Aldrich, subsidiary of Merck KGaA, Darmstadt, Germany; DW/EtOH) were used. Folin–Ciocalteu reagent (Merck, Darmstadt, Germany), Na_2_CO_3_ and K_2_S_2_O_8_ (Sigma Aldrich, subsidiary of Merck KGaA, Darmstadt, Germany), gallic acid (GA, Sigma Aldrich, subsidiary of Merck KGaA, Darmstadt, Germany), 2,2′-azinobis 3-ethylbenzothiazoline-6-sulfonate (ABTS) and Trolox (Merck, Darmstadt, Germany) were used to evaluate the phenolic content and antioxidant activity of the obtained eucalyptus extracts.

### 3.2. Extraction

Before extraction, dried eucalyptus leaves were very finely ground to facilitate the contact between the plant material and solvents. Plant material/solvent mass ratio was 1/10 regardless of the extraction medium or the method used. The extraction experiments are summarized in Table 3.

Extraction by magnetic stirring (MS). Plant material and extraction medium (DW or DW/EtOH) were subjected to magnetic stirring at RT (~25 °C) and protected from light, to prevent the degradation of photo- and thermo-sensitive components. The process was carried out for 10/20/30 min, using a Heidolph shaker plate (1000 RPM). From this work sequence, 6 eucalyptus extract samples resulted (experiments 1–6).

Extraction by ultrasound treatment (US). Extraction was performed by using an Elma ultrasonic bath (model S 10/H) with a power of 30 W. The process was carried out for 10/20/30 min, protected from light, obtaining 6 samples of eucalyptus extracts (experiments 7–12). For these experiments, an ice bath was used to prevent the heating of the extraction mixture.

Microwave-assisted extraction (MW). A household microwave device (Hansa brand, part of Amica Group, Poland) was used for these experiments. There were 3 extraction times (10/20/30 s) tested on 2 power levels (360 W/600 W). From this work sequence, 12 eucalyptus extract samples were obtained (experiments 13–24).

All the resulting extract samples were filtered (Whatman filter paper no. 1), separating the liquid part, and further conditioned by lyophilization.

### 3.3. Characterization

Dry matter fraction. The amount of dry matter was determined by lyophilization of the obtained extracts. For the extracts obtained in the hydroalcoholic solvent, first the ethanol was removed by evaporation under reduced pressure. Subsequently, eucalyptus extracts were frozen at approximately −40 °C (72 h) and then lyophilized for 28 h, using an Alpha 1–2 LSC basic lyophilizer (Martin Christ, Osterode, Germany). Dry extracts (expressed as dry matter, DM) were stored at approx. −18 °C, protected from light.

Extract processing. For evaluation, lyophilized extract samples were “reconstituted” by solubilization in the extraction solvent (DW or DW/EtOH mixture), to a final concentration of 10 mg/mL.

Quantification of total phenolic content (TPC). This was performed using the adapted Folin–Ciocalteu method [28,29,30]. Briefly, 0.01 mL eucalyptus extract, 5 mL Folin–Ciocalteu reagent and 0.99 mL DW were homogenized and kept at RT, in a dark place, for 10 min. Then, 4 mL Na_2_CO_3_ solution (20%) was added. Samples were kept at RT, protected from light, for a total incubation time of 2 h, to complete the reaction and for the blue detectable in the UV–Vis range complex to be generated. In the same way, standard solutions were prepared, replacing the eucalyptus extract with gallic acid solution of known concentration (0.5–50 mg/mL). The absorbances of the test samples (eucalyptus extracts) and standard samples were measured, at 760 nm, using a Helios Beta UV–Vis spectrophotometer (Thermo Electron Corporation, Waltham, MA, USA). TPC was expressed as mg gallic acid equivalents/g dry matter (mg GAE/g DM). Measurements were performed in triplicate; the obtained results are expressed as mean ± standard deviation.

Evaluation of antioxidant activity (AA). This was performed by means of the widely used TEAC (Trolox Equivalent Antioxidant Capacity) method [49]. In brief, the TEAC method is based on the decrease in the absorption of the cationic radical ABTS^+^• in the presence of an antioxidant. The generation of the ABTS^+^• radical was achieved by the reaction of ABTS with K_2_S_2_O_8_, resulting in a stable, blue-green radical that absorbs strongly in the visible spectrum. The presence of antioxidant molecules reduces the ABTS^+^• radical, leading to a discoloration of the solution and, therefore, to a decrease in absorption. This decrease in absorption was determined by spectrophotometric measurements at 734 nm. Standard stock solution of Trolox was used, diluted to a concentration range of 0.5–2.5 mM.

Spectrophotometric measurements were performed as follows: an exact volume of 990 μL of the ABTS•+ stock solution was introduced into a cuvette, and the initial optical density (OD_i_) was immediately recorded. Subsequently, 10 μL of the Trolox solution was added to the same cuvette and, after exactly 1 min, the final optical density (OD_f_) was recorded. The same procedure was followed for all standard Trolox samples (0.5–2.5 mM). For the eucalyptus extracts, the same protocol was applied, replacing the Trolox solutions with 10 μL of each extract (10 mg/mL concentration). OD_i_ and OD_f_ were used to determine the percentage of inhibition (I%), according to Equation (1):

(1) $$I(\%)=\frac{OD_{i}-OD_{f}}{OD_{i}}\times 100$$

All measurements were performed against ethanol (blank sample), using a SHIMADZU UV–Vis spectrophotometer (Shimadzu Corporation, Kyoto, Japan), model UVmini-1240. The calculated inhibition percentage (I%) is correlated with the antioxidant capacity, which is calculated based on the Trolox solution calibration curve. Determination of antioxidant activity was performed in triplicate and results are expressed as mean ± standard deviation. The AA values of the extracts were expressed as Trolox equivalents/g DM (mg TE/g DM).

Phytochemical profile. This was evaluated by high-resolution mass spectrometry analysis (FT-ICR MS), performed by using a Fourier Transform-ion cyclotron resonance (FT-ICR) spectrometer, SolariX XR 15T (Bruker Daltonics, Bremen, Germany). The sample was introduced by direct infusion. Positive ESI ionization was performed with a sample flow rate of 270 µL/h, with a nebulization gas pressure (N_2_) of 1.2 bar at 300 °C and a flow rate of 1.5 L/min. Negative ESI ionization was performed with a sample flow rate of 120 µL/h, with a nebulization gas pressure (N_2_) of 1.2 bar at 300 °C and a flow rate of 1.8 L/min. The spectra were recorded over a mass range between 92 and 1500 amu at a source voltage of 4500 V.

Estimation of the specific energy consumption (SEC_est_, kWh/g GAE). This was calculated for the involved extraction techniques by using Equation (2), based on the estimated operational energy demand per unit mass of recovered phenolic compounds, expressed as GAE equivalents [48].

(2) $${SEC}_{est}=\frac{E\times 1000}{TPC\times {DM}_{extract}}$$

where E is the estimated operational energy demand (E = P × t), calculated from the nominal equipment power and extraction time, TPC is the total phenolic content expressed as mg GAE/g DM, and DM_extract_ represents the dry mass of the recovered extract (g).

## 4. Conclusions

This research study investigated the influence of extraction method, solvent system, and processing time on the recovery of phenolic compounds and antioxidant activity of *Eucalyptus globulus* leaf extracts. The obtained results highlighted the important role of both extraction technique and solvent composition on phenolic species recovery under the investigated experimental conditions.

Hydroalcoholic extraction systems (DW/EtOH, 80/20 *v*/*v*), combined with non-conventional extraction methods—MW and US—generally promoted higher TPC values compared to aqueous extraction. MW (maximum 30 s) conduces to extracts with TPC values up to ~3-fold (≈200–230%) higher than magnetic stirring (30 min), while US provided phenolic contents approximately 1.5–2 times higher than the reference method. From the involved experimental conditions, MW extraction favored rapid recovery of phenolic compounds during very short processing times, while US extraction provided a more gradual extraction profile associated with comparatively higher TPC/DM values under prolonged processing conditions.

The antioxidant activity results showed only a moderate proportional relationship with total phenolic content, suggesting that antioxidant behavior may also be influenced by the qualitative composition of the extracts, the relative distribution of phenolic classes, and the stability of antioxidant compounds during extraction. Important to note is the higher antioxidant activity for the extracts obtained by microwave extraction at 360 W and not for those obtained when the process was carried out at a higher power (600 W). This finding suggests a possible degradation of some antioxidant species under higher energy input. Therefore, to prevent the alteration of sensitive chemical compounds, a moderate microwave power is recommended.

FT-ICR MS analysis indicated similar qualitative phytochemical profiles among the investigated extracts, dominated by phenolic acids, flavonoids, and ellagitannins, indicating that the used parameters are mild and do not alter the chemical profile of the plant extracts. However, the phytochemical assignments should be interpreted as tentative compositional annotations based on accurate mass measurements.

Overall, the investigated extraction conditions highlighted the potential of non-conventional extraction approaches for the recovery of phenolic compounds from commercially available eucalyptus leaves under relatively mild extraction conditions. Nevertheless, additional studies concerning extract stability, safety, bioactivity, and formulation compatibility are necessary before considering consistent practical cosmetic, nutraceutical, or pharmaceutical applications.

## Figures and Tables

**Figure 6:**
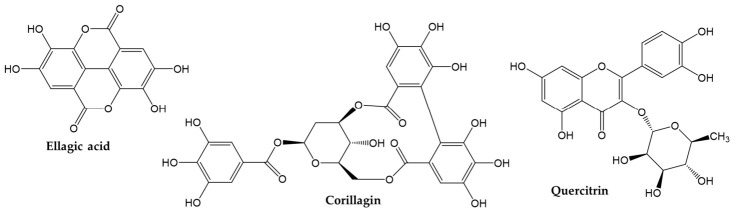
Chemical structures of representative phenolic compounds tentatively assigned in the composition of *Eucalyptus globulus* extracts.

**Figure 7:**
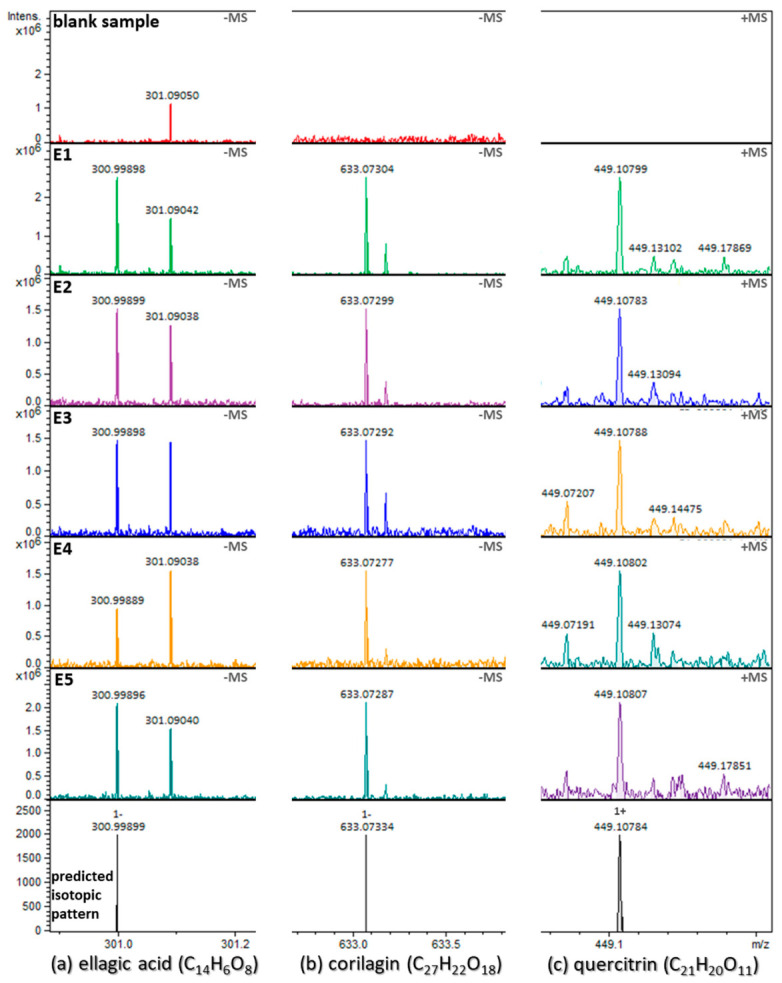
Representative FT-ICR mass spectra of selected phenolic compounds assigned for the studied eucalyptus extracts, with characteristic ion signals for a phenolic acid (ellagic acid, ESI−), an ellagitannin (corilagin, ESI−), and a flavonoid (quercitrin, ESI+). Spectra are shown for the blank sample, extracts E1–E5 and predicted isotopic patterns, presented for qualitative compositional comparison only.

**Table 1:** Selected eucalyptus extracts analyzed by FT-ICR MS and their acronyms.

Extract Sample Analyzed by FT-ICR	The Extract Acronym *
MW 360 W_30 s (DW/EtOH)	E1
MW 600 W_30 s (DW/EtOH)	E2
US_30 min (DW-EtOH)	E3
US_30 min (DW)	E4
MW 360 W_30 s (DW)	E5

* Acronyms used only in the context of the phytochemical profile.

**Table 2:** Main phenolic compounds tentatively assigned in eucalyptus extracts by FT-ICR MS [12,38,45,46].

Class	Compound	Molecular Formula	Ionization Mode	Detected in Samples
Phenolic acids	ellagic acid	C_14_H_6_O_8_	ESI−/ESI+	E1–E5
	brevifolin carboxylic acid	C_13_H_8_O_8_	ESI+	E1–E5
	salicylic acid β-D-glucuronide	C_13_H_14_O_9_	ESI+	E1–E5
	grandinol	C_13_H_16_O_5_	ESI+	E1–E5
	chlorogenic acid	C_16_H_18_O_9_	ESI+	E1–E5
Galloyl and ellagic acid derivatives	galloyl glucose	C_13_H_16_O_10_	ESI−/ESI+	E1–E5
	digalloyl glucose	C_20_H_20_O_14_	ES−/ESI+	E1–E5
	methylellagic derivatives	C_15_H_8_O_8_	ESI+	E1–E5
Flavonoids	quercetin derivative	C_21_H_18_O_12_	ESI+	E1–E5
	quercetin derivative	C_21_H_18_O_13_	ESI−/ESI+	E1–E5
	quercitrin	C_21_H_20_O_11_	ESI+	E1–E5
	isoquercitrin	C_21_H_20_O_12_	ESI+	E1–E5
	catechin/epicatechin-type flavonoid	C_15_H_14_O_6_	ESI+	E1–E5
Hydrolysable tannins (ellagitannins)	corilagin	C_27_H_22_O_18_	ESI−/ESI+	E1–E5
	pedunculagin	C_34_H_24_O_22_	ESI−/ESI+	E1–E5
	tellimagrandin I	C_34_H_26_O_22_	ESI−/ESI+	E1–E5
	dimethylellagic glucoside	C_22_H_20_O_13_	ESI+	E1–E5
	cypellocarpin C	C_26_H_32_O_11_	ESI+	E1–E5
	hydrolizable tannins-related	C_21_H_10_O_13_	ESI−/ESI+	E1–E5
	hydrolizable tannins-related	C_14_H_10_O_10_	ESI+	E1–E5
Other compounds (phenolic or non-phenolic)	quinic acid	C_7_H_12_O_6_	ESI−/ESI+	E1–E5
	unassigned	C_31_H_34_O_14_	ESI+	E1–E5
	unassigned	C_28_H_24_O_16_	ESI+	E1–E5

**Table 3:** The extraction experiments carried out within the study.

Extraction	Extraction Solvent	Extraction Parameters
Experiments 1–3	DW	magnetic stirring 10/20/30 min; RT; 1000 RPM
Experiments 4–6	DW/EtOH	
Experiments 7–9	DW	ultrasound 10/20/30 min; ice bath; 30 W
Experiments 10–12	DW/EtOH	
Experiments 13–15	DW	microwaves 10/20/30 s; RT; 360 W
Experiments 16–18	DW/EtOH	
Experiments 19–21	DW	microwaves 10/20/30 s; RT; 600 W
Experiments 22–24	DW/EtOH	

## Data Availability

The raw data supporting the conclusions of this article will be made available by the authors on request.

## Supplementary Materials

The following supporting information can be downloaded at https://www.mdpi.com/article/10.3390/molecules31111927/s1, Figure S1. FT-ICR MS spectrum of ellagic acid (C_14_H_6_O_8_) detected in eucalyptus extract (E1–E5) in negative ionization mode (ESI−). The deprotonated molecular ion [M−H]^−^ at *m*/*z* ~301 is highlighted. Figure S2. FT-ICR MS spectrum of ellagic acid (C_14_H_6_O_8_) detected in eucalyptus extract (E1–E5) in positive ionization mode (ESI+). The protonated molecular ion [M+H]^+^ at *m*/*z* ~303 is highlighted. Figure S3. FT-ICR MS spectrum of brevifolin carboxylic acid (C_13_H_8_O_8_) detected in eucalyptus extract (E1–E5) in positive ionization mode (ESI+). The protonated molecular ion [M+H]^+^ at *m*/*z* ~293 is highlighted. Figure S4. FT-ICR MS spectrum of salicylic acid β-D-glucuronide (C_13_H_14_O_9_) detected in eucalyptus extract (E1–E5) in positive ionization mode (ESI+). The protonated molecular ion [M+H]^+^ at *m*/*z* ~315 is highlighted. Figure S5. FT-ICR MS spectrum of grandinol (C_13_H_16_O_5_) detected in eucalyptus extract (E1–E5) in positive ionization mode (ESI+). The protonated molecular ion [M+H]^+^ at *m*/*z* ~253 is highlighted. Figure S6. FT-ICR MS spectrum of chlorogenic acid (C_16_H_18_O_9_) detected in eucalyptus extract (E1–E5) in positive ionization mode (ESI+). The protonated molecular ion [M+H]^+^ at *m*/*z* ~355 is highlighted. Figure S7. FT-ICR MS spectrum of galloyl glucose (C_13_H_16_O_10_) detected in eucalyptus extract (E1–E5) in negative ionization mode (ESI−). The deprotonated molecular ion [M−H]^−^ at *m*/*z* ~331 is highlighted. Figure S8. FT-ICR MS spectrum of galloyl glucose (C_13_H_16_O_10_) detected in eucalyptus extract (E1–E5) in positive ionization mode (ESI+). The protonated molecular ion [M+H]^+^ at *m*/*z* ~333 is highlighted. Figure S9. FT-ICR MS spectrum of digalloyl glucose (C_20_H_20_O_14_) detected in eucalyptus extract (E1–E5) in negative ionization mode (ESI−). The deprotonated molecular ion [M−H]^−^ at *m*/*z* ~483 is highlighted. Figure S10. FT−ICR MS spectrum of digalloyl glucose (C_20_H_20_O_14_) detected in eucalyptus extract (E1–E5) in positive ionization mode (ESI+). The protonated molecular ion [M+H]^+^ at *m*/*z* ~485 is highlighted. Figure S11. FT-ICR MS spectrum of a methylellagic derivative (C_15_H_8_O_8_) detected in eucalyptus extract (E1–E5) in positive ionization mode (ESI+). The protonated molecular ion [M+H]^+^ at *m*/*z* ~317 is highlighted. Figure S12. FT-ICR MS spectrum of a quercetin derivative (C_21_H_18_O_12_) detected in eucalyptus extract (E1–E5) in positive ionization mode (ESI+). The protonated molecular ion [M+H]^+^ at *m*/*z* ~463 is highlighted. Figure S13. FT-ICR MS spectrum of a quercetin derivative (C_21_H_18_O_13_) detected in eucalyptus extract (E1–E5) in negative ionization mode (ESI−). The deprotonated molecular ion [M+H]^−^ at *m*/*z* ~477 is highlighted. Figure S14. FT-ICR MS spectrum of a quercetin derivative (C_21_H_18_O_13_) detected in eucalyptus extract (E1–E5) in positive ionization mode (ESI+). The protonated molecular ion [M+H]^+^ at *m*/*z* ~479 is highlighted. Figure S15. FT-ICR MS spectrum of quercitrin (C_21_H_20_O_11_) detected in eucalyptus extract (E1–E5) in positive ionization mode (ESI+). The protonated molecular ion [M+H]^+^ at *m*/*z* ~521 is highlighted. Figure S16. FT-ICR MS spectrum of isoquercitrin (C_21_H_20_O_12_) detected in eucalyptus extract (E1–E5) in positive ionization mode (ESI+). The protonated molecular ion [M+H]^+^ at *m*/*z* ~465 is highlighted. Figure S17. FT-ICR MS spectrum o catechin/epicatechin flavonoid (C_15_H_14_O_6_) detected in eucalyptus extract (E1–E5) in positive ionization mode (ESI+). The protonated molecular ion [M+H]^+^ at *m*/*z* ~291 is highlighted. Figure S18. FT-ICR MS spectrum of corilagin (C_27_H_22_O_18_) detected in eucalyptus extract (E1–E5) in negative ionization mode (ESI−). The deprotonated molecular ion [M+H]^−^ at *m*/*z* ~633 is highlighted. Figure S19. FT-ICR MS spectrum of corilagin (C_27_H_22_O_18_) detected in eucalyptus extract (E1–E5) in positive ionization mode (ESI+). The protonated molecular ion [M+H]^+^ at *m*/*z* ~635 is highlighted. Figure S20. FT-ICR MS spectrum of pedunculagin (C_34_H_24_O_22_) detected in eucalyptus extract (E1–E5) in negative ionization mode (ESI−). The deprotonated molecular ion [M+H]^−^ at *m*/*z* ~783 is highlighted. Figure S21. FT-ICR MS spectrum of pedunculagin (C_34_H_24_O_22_) detected in eucalyptus extract (E1–E5) in positive ionization mode (ESI+). The protonated molecular ion [M+H]^+^ at *m*/*z* ~785 is highlighted. Figure S22. FT-ICR MS spectrum of tellimagrandin I (C_34_H_26_O_22_) detected in eucalyptus extract (E1–E5) in positive ionization mode (ESI+). The protonated molecular ion [M+H]^+^ at *m*/*z* ~787 is highlighted. Figure S23. FT-ICR MS spectrum of a dimethyl ellagic acid glycoside (C_22_H_20_O_13_) detected in eucalyptus extract (E1–E5) in positive ionization mode (ESI+). The protonated molecular ion [M+H]^+^ at *m*/*z* ~493 is highlighted. Figure S24. FT-ICR MS spectrum of cypellocarpin C (C_26_H_32_O_11_) detected in eucalyptus extract (E1–E5) in positive ionization mode (ESI+). The protonated molecular ion [M+H]^+^ at *m*/*z* ~521 is highlighted. Figure S25. FT-ICR MS spectrum of a hydrolizable tannins-related molecule (C_21_H_10_O_13_) detected in eucalyptus extract (E1–E5) in negative ionization mode (ESI−). The deprotonated molecular ion [M+H]^−^ at *m*/*z* ~469 is highlighted. Figure S26. FT-ICR MS spectrum of a hydrolizable tannins-related molecule (C_21_H_10_O_13_) detected in eucalyptus extract (E1–E5) in positive ionization mode (ESI+). The protonated molecular ion [M+H]^+^ at *m*/*z* ~471 is highlighted. Figure S27. FT-ICR MS spectrum of a hydrolizable tannins-related molecule (C_14_H_10_O_10_) detected in eucalyptus extract (E1–E5) in positive ionization mode (ESI+). The protonated molecular ion [M+H]^+^ at *m*/*z* ~339 is highlighted. Figure S28. FT-ICR MS spectrum of quinic acid (C_7_H_12_O_6_) detected in eucalyptus extract (E1–E5) in negative ionization mode (ESI−). The deprotonated molecular ion [M+H]^−^ at *m*/*z* ~191 is highlighted. Figure S29. FT-ICR MS spectrum of quinic acid (C_7_H_12_O_6_) detected in eucalyptus extract (E1–E5) in positive ionization mode (ESI+). The protonated molecular ion [M+H]^+^ at *m*/*z* ~193 is highlighted. Figure S30. FT-ICR MS spectrum of unassigned compound (C_31_H_34_O_14_) detected in eucalyptus extract (E1–E5) in positive ionization mode (ESI+). The protonated molecular ion [M+H]^+^ at *m*/*z* ~631 is highlighted. Figure S31. FT-ICR MS spectrum of unassigned compound (C_28_H_24_O_16_) detected in eucalyptus extract (E1–E5) in positive ionization mode (ESI+). The protonated molecular ion [M+H]^+^ at *m*/*z* ~617 is highlighted. Figure S32. Estimated specific energy demand related to phenolic species recovery from *Eucalyptus globulus* leaves.
