# Supplementary material for: From Conventional to Sustainable Extraction: Improving Phenolic Species Recovery from Eucalyptus globulus Leaves
Source: Molecules. 2026 Jun 3;31(11):1927. doi: 10.3390/molecules31111927 (PMC13258619; doi:10.3390/molecules31111927)
Supplement: Supplementary file 1 [file molecules-31-01927-s001.zip › molecules-4325479-supplementary.pdf]

molecules

Supplementary Materials

From conventional to sustainable extraction: Improving phenolic species recovery from *Eucalyptus globulus* leaves

Cristina Ott, Raluca Stan, Mihaela Tociu, Alina Morosan and Brindusa Balanuca *

Department of Organic Chemistry "C. Nenitescu", Faculty of Chemical Engineering and Biotechnology, National University of Science and Technology POLITEHNICA Bucharest, Bucharest, 011061, Romania; cristina.ott@upb.ro (C.O.); raluca.stan@upb.ro (R.S.); mihaela.tociu@upb.ro (M.T.); alina.morosan@upb.ro (A.M.); brindusa.balanuca@upb.ro (B.B.)

* Correspondence: brindusa.balanuca@upb.ro (B.B.)

The following figures support the experimental data presented:

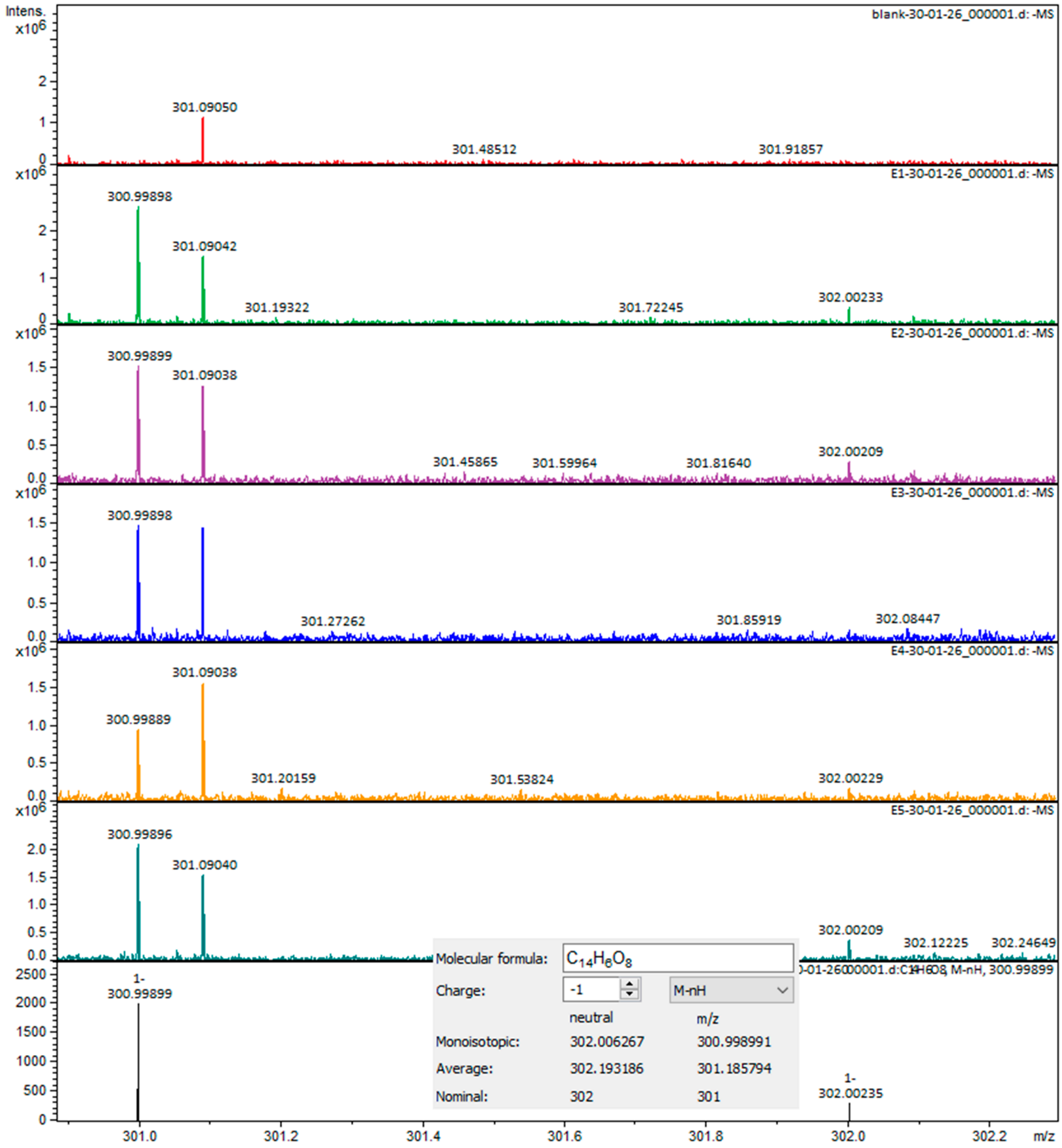

Figure S1. FT-ICR MS spectrum of ellagic acid ($C_{14}H_6O_8$) detected in eucalyptus extract (E1-E5) in negative ionization mode (ESI-). The deprotonated molecular ion $[M–H]^-$ at m/z ~301 is highlighted.

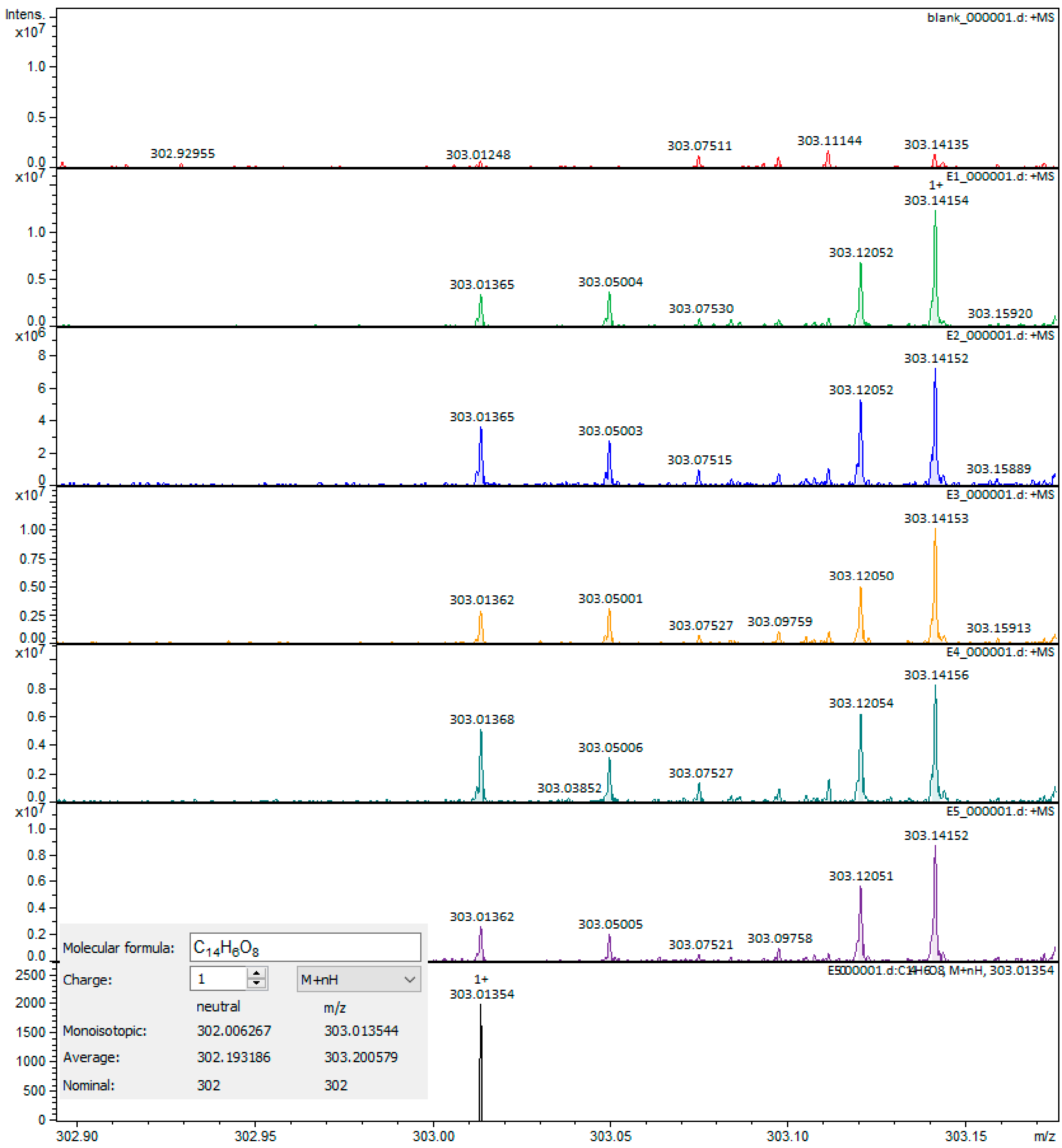

Figure S2. FT-ICR MS spectrum of ellagic acid ($C_{14}H_6O_8$) detected in eucalyptus extract (E1-E5) in positive ionization mode (ESI+). The protonated molecular ion $[M+H]^+$ at m/z ~303 is highlighted.

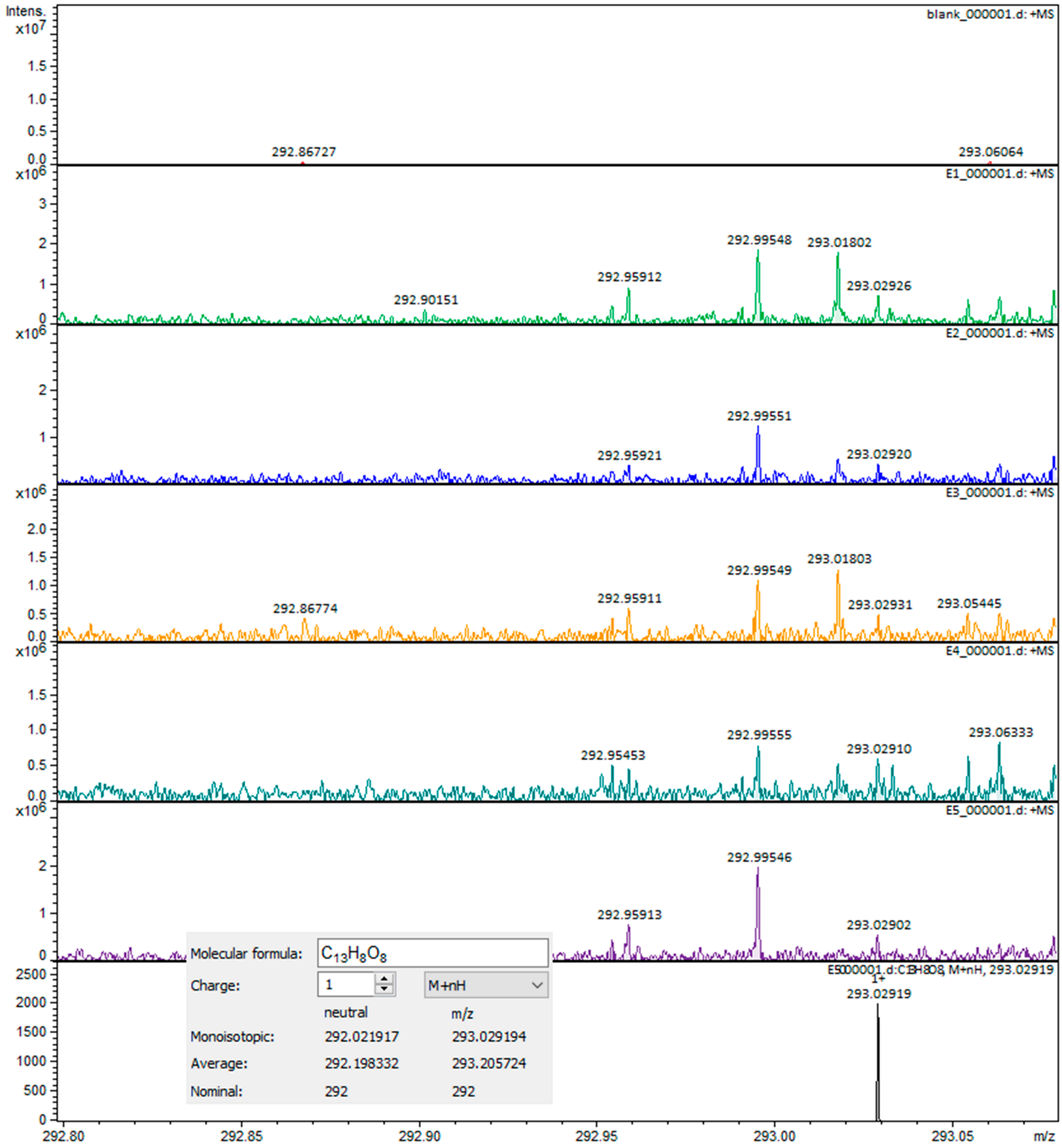

Figure S3. FT-ICR MS spectrum of brevifolin carboxylic acid ($C_{13}H_8O_8$) detected in eucalyptus extract (E1-E5) in positive ionization mode (ESI+). The protonated molecular ion $[M+H]^+$ at m/z ~293 is highlighted.

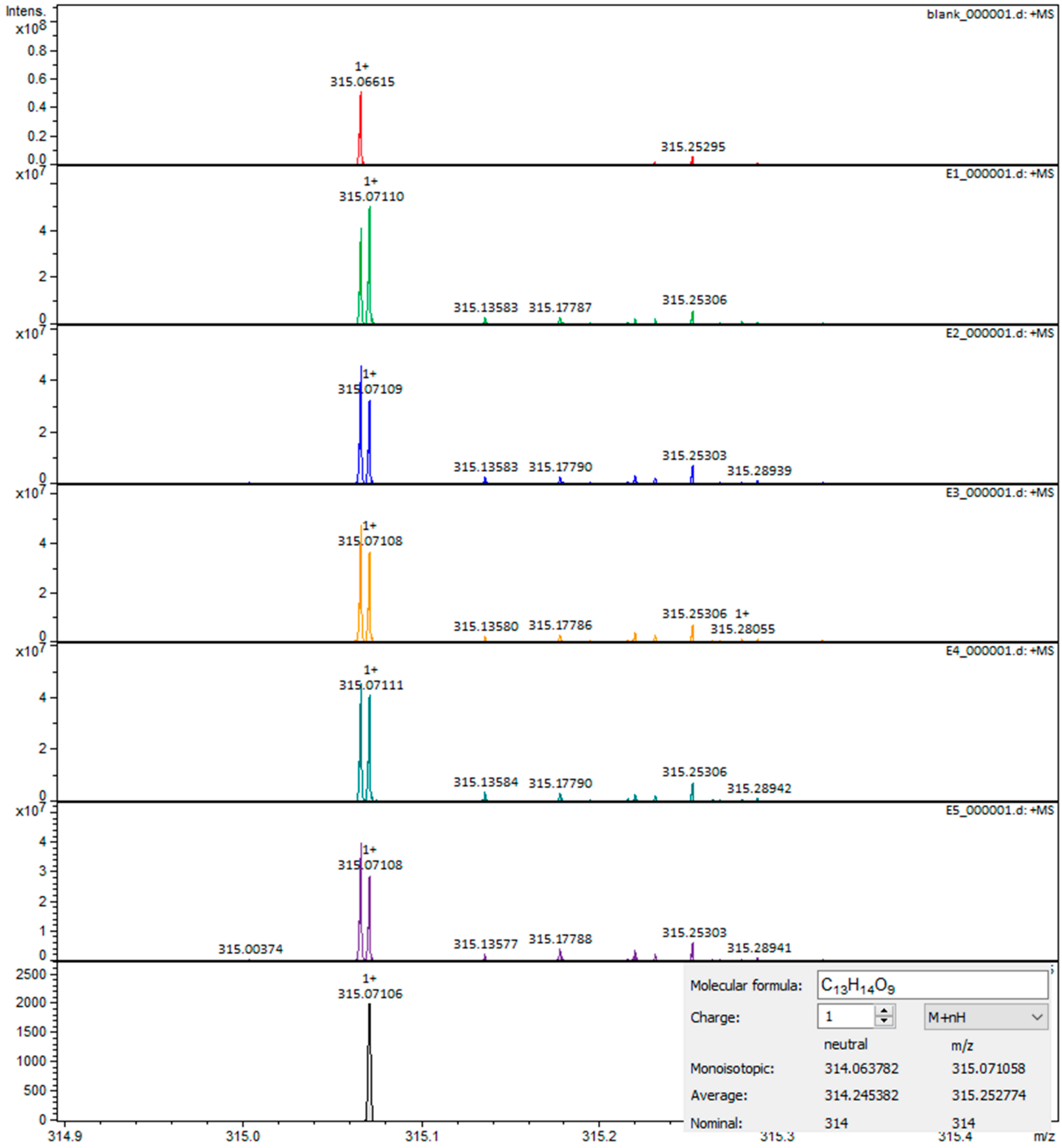

Figure S4. FT-ICR MS spectrum of salicylic acid β-D-glucuronide ($C_{13}H_{14}O_9$) detected in eucalyptus extract (E1-E5) in positive ionization mode (ESI+). The protonated molecular ion $[M+H]^+$ at m/z ~315 is highlighted.

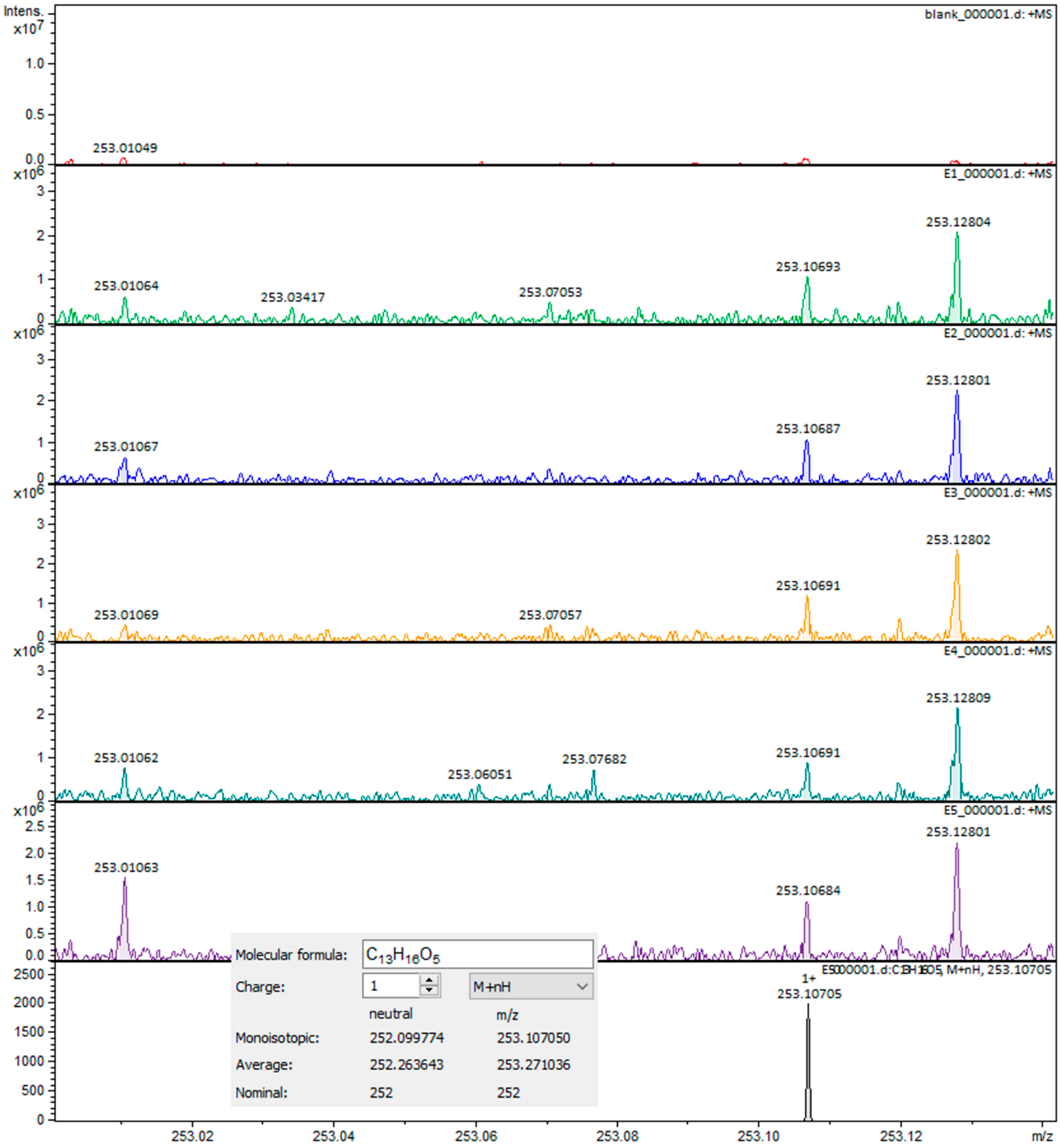

Figure S5. FT-ICR MS spectrum of grandinol ($C_{13}H_{16}O_5$) detected in eucalyptus extract (E1-E5) in positive ionization mode (ESI+). The protonated molecular ion $[M+H]^+$ at m/z ~253 is highlighted.

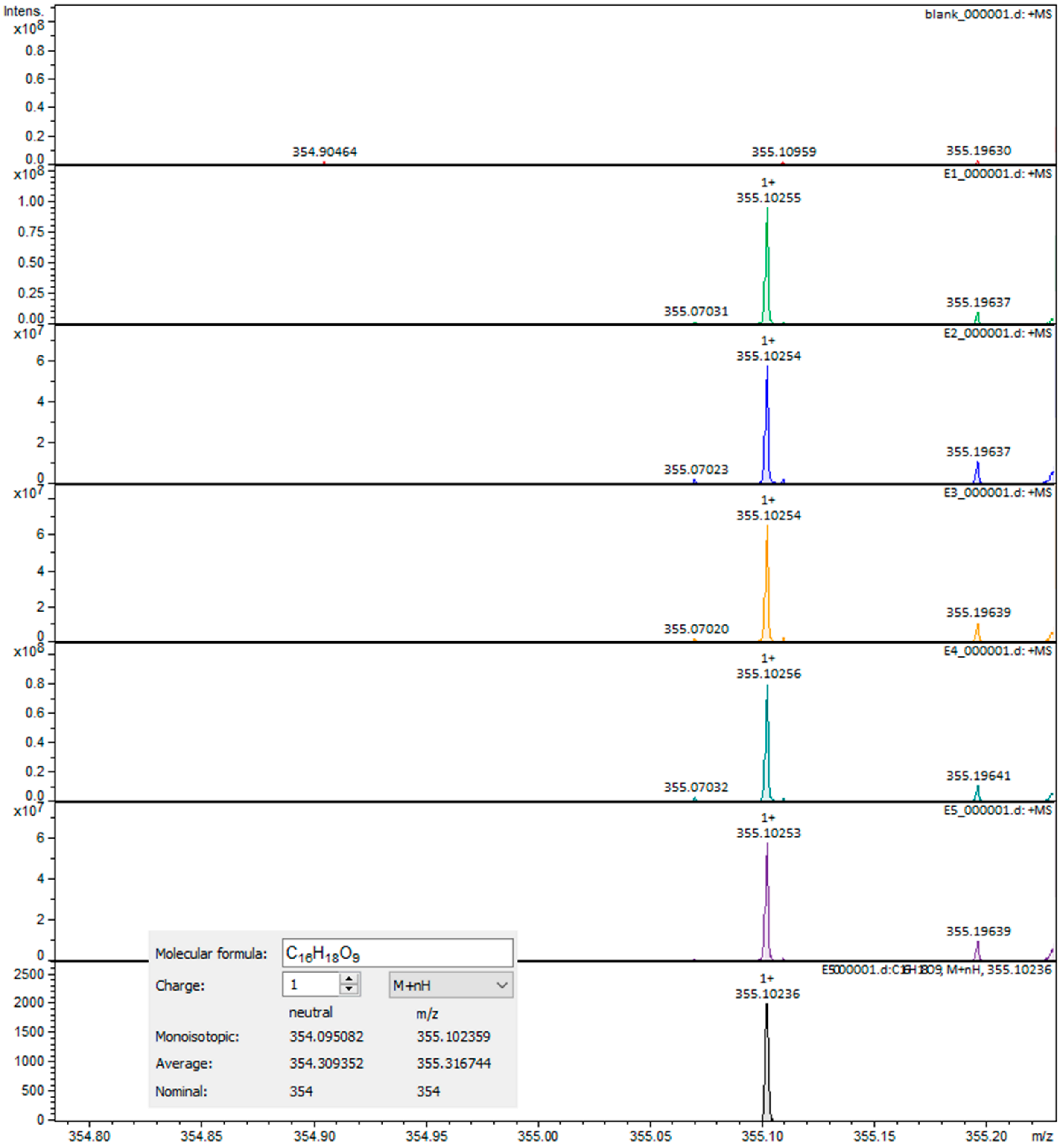

Figure S6. FT-ICR MS spectrum of chlorogenic acid ($C_{16}H_{18}O_9$) detected in eucalyptus extract (E1-E5) in positive ionization mode (ESI+). The protonated molecular ion $[M+H]^+$ at m/z ~355 is highlighted.

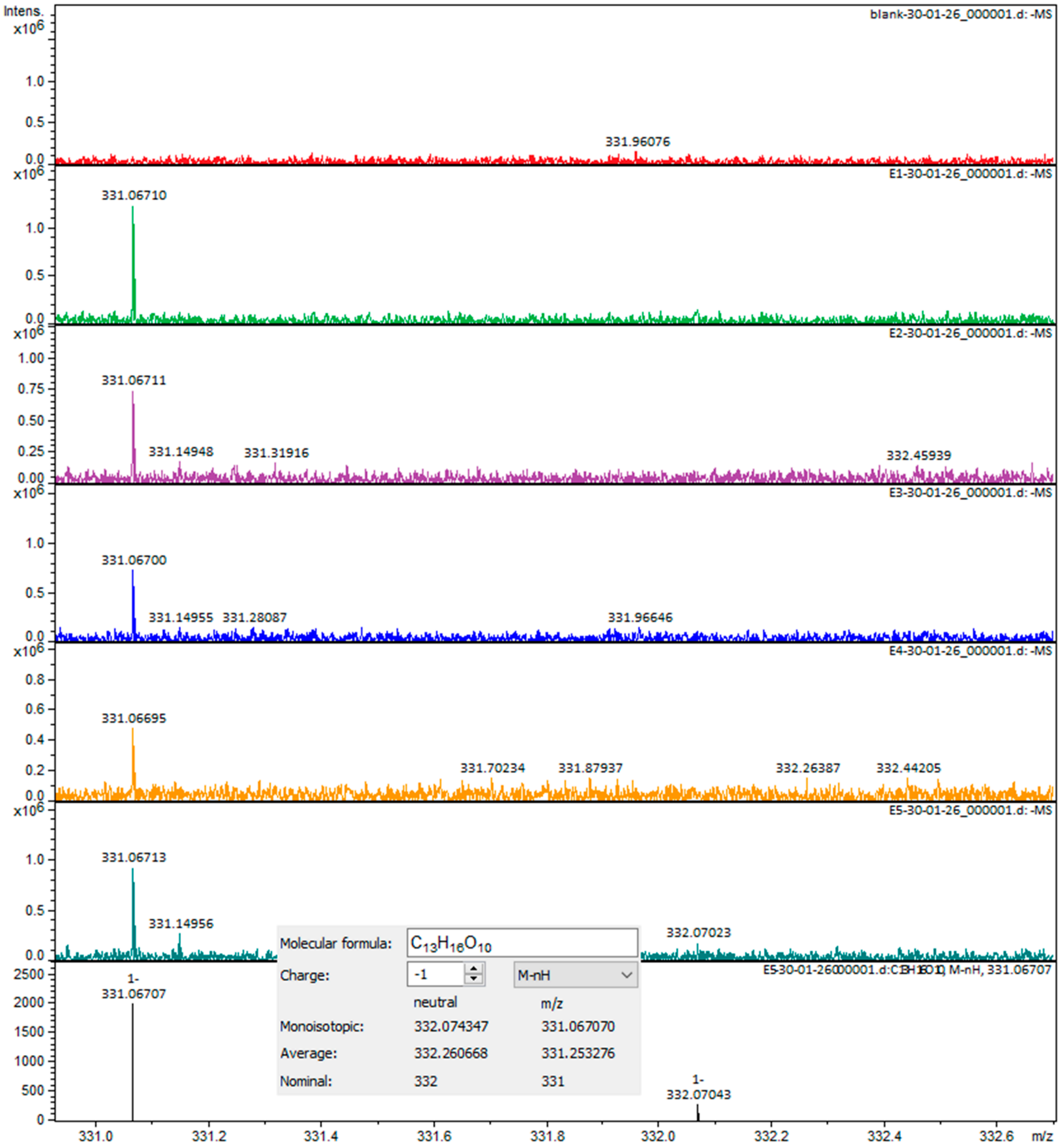

Figure S7. FT-ICR MS spectrum of galloyl glucose ($C_{13}H_{16}O_{10}$) detected in eucalyptus extract (E1-E5) in negative ionization mode (ESI-). The deprotonated molecular ion $[M–H]^-$ at m/z ~331 is highlighted.

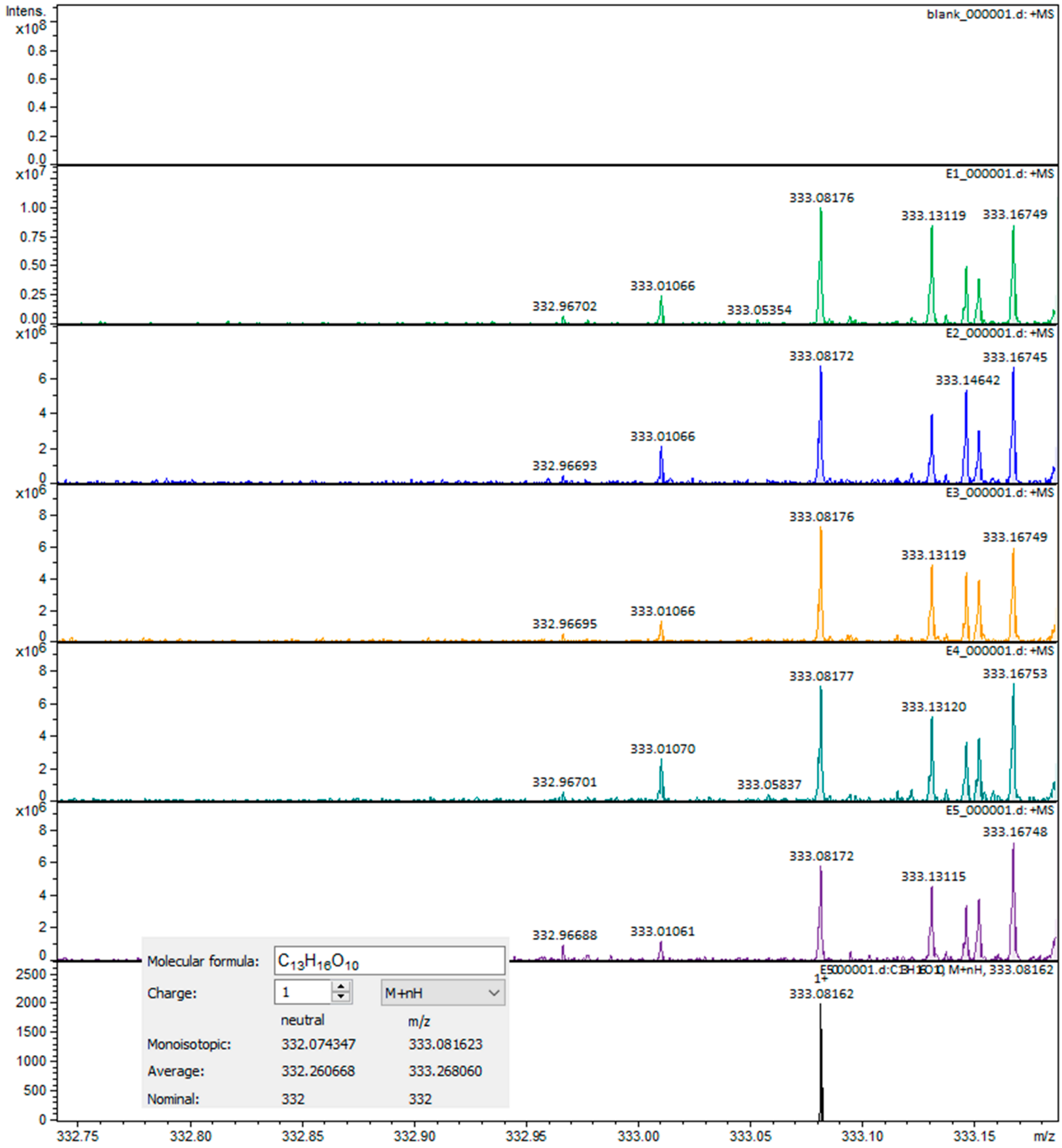

Figure S8. FT-ICR MS spectrum of galloyl glucose ($C_{13}H_{16}O_{10}$) detected in eucalyptus extract (E1-E5) in positive ionization mode (ESI+). The protonated molecular ion $[M+H]^+$ at m/z ~333 is highlighted.

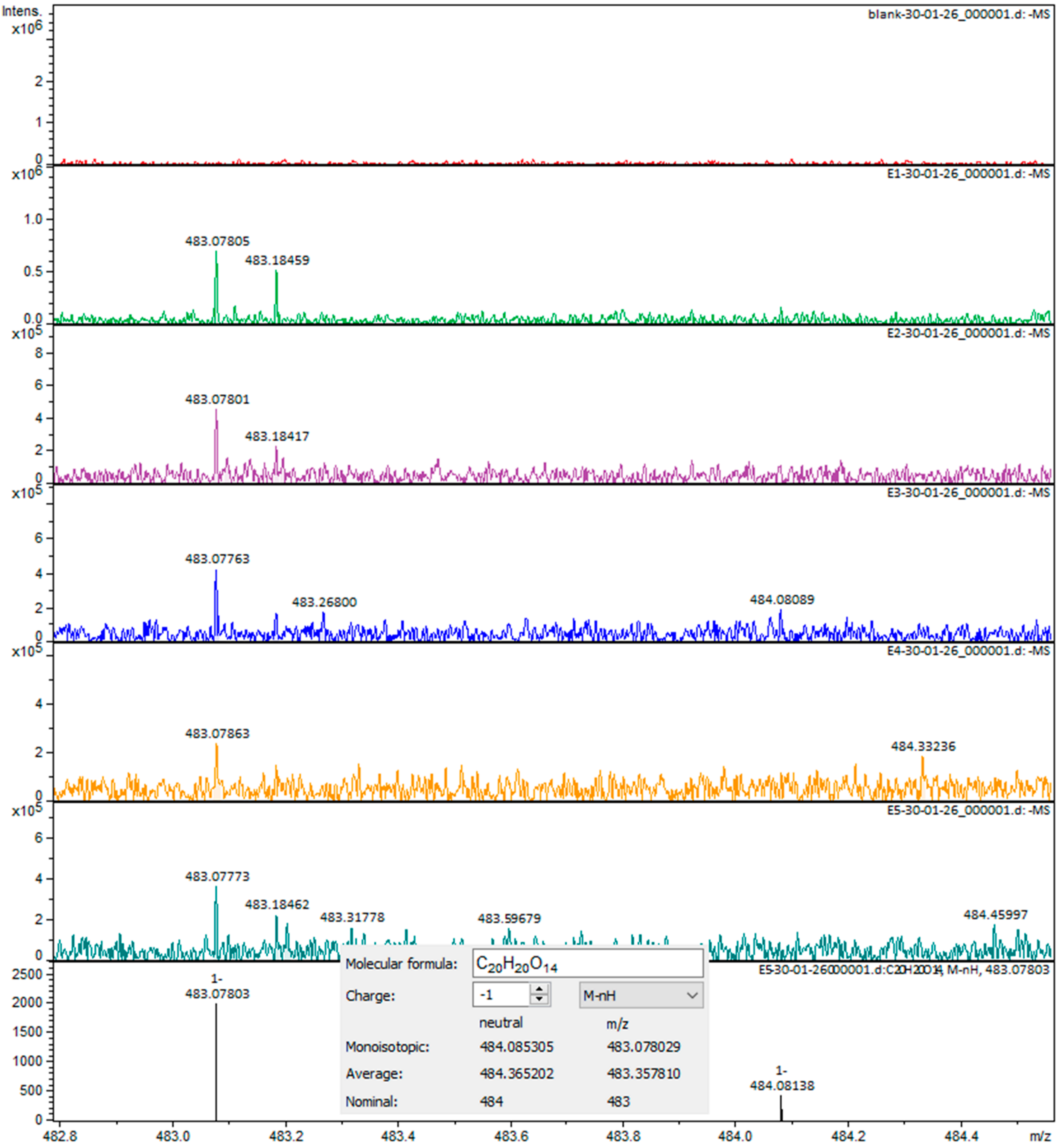

Figure S9. FT-ICR MS spectrum of digalloyl glucose ($C_{20}H_{20}O_{14}$) detected in eucalyptus extract (E1-E5) in negative ionization mode (ESI-). The deprotonated molecular ion $[M–H]^-$ at m/z ~483 is highlighted.

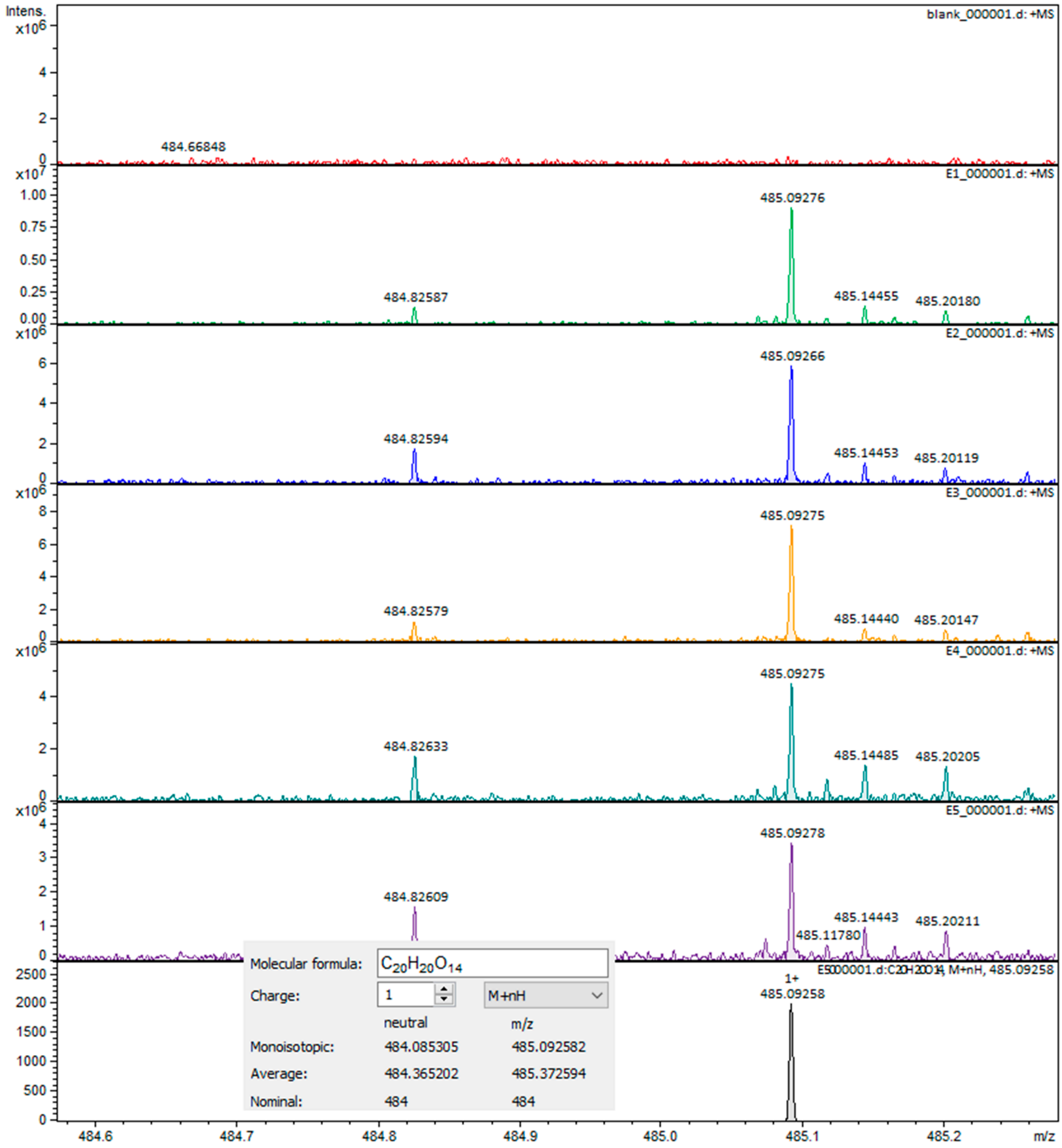

Figure S10. FT-ICR MS spectrum of digalloyl glucose ($C_{20}H_{20}O_{14}$) detected in eucalyptus extract (E1-E5) in positive ionization mode (ESI+). The protonated molecular ion $[M+H]^+$ at m/z ~485 is highlighted.

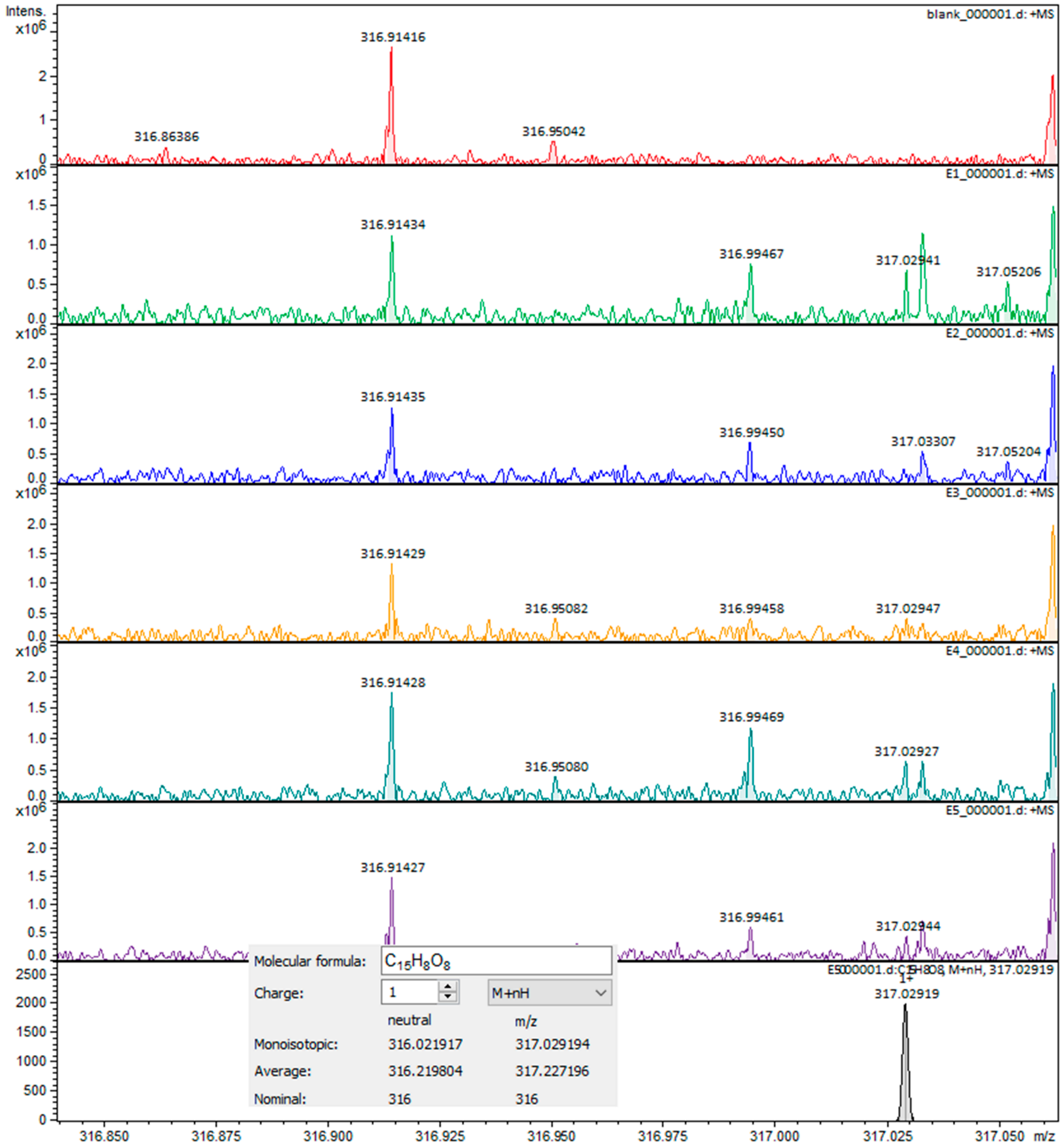

Figure S11. FT-ICR MS spectrum of a methylellagic derivative ($C_{15}H_8O_8$) detected in eucalyptus extract (E1-E5) in positive ionization mode (ESI+). The protonated molecular ion $[M+H]^+$ at m/z ~317 is highlighted.

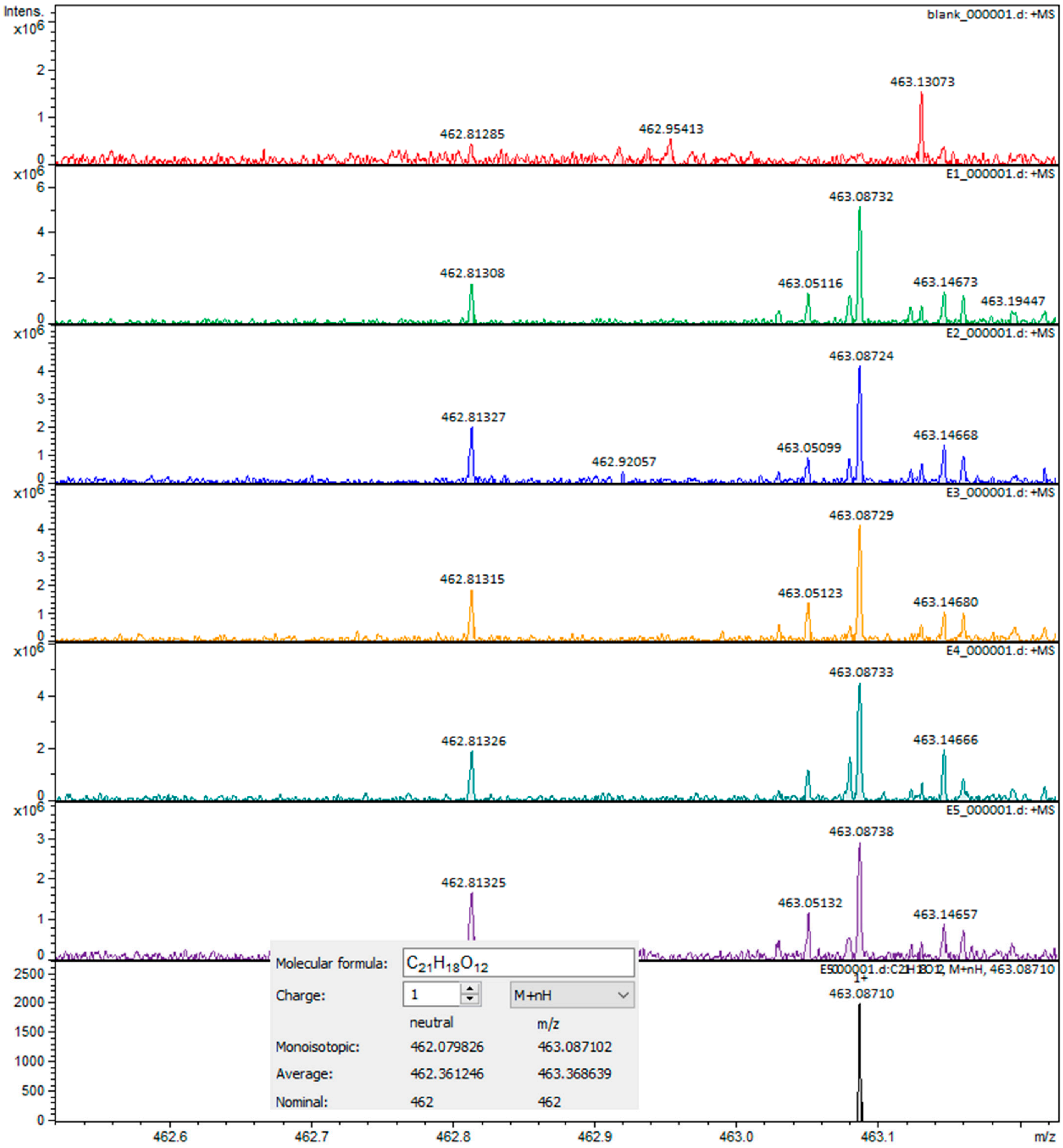

Figure S12. FT-ICR MS spectrum of a quercetin derivative ($C_{21}H_{18}O_{12}$) detected in eucalyptus extract (E1-E5) in positive ionization mode (ESI+). The protonated molecular ion $[M+H]^+$ at m/z ~463 is highlighted.

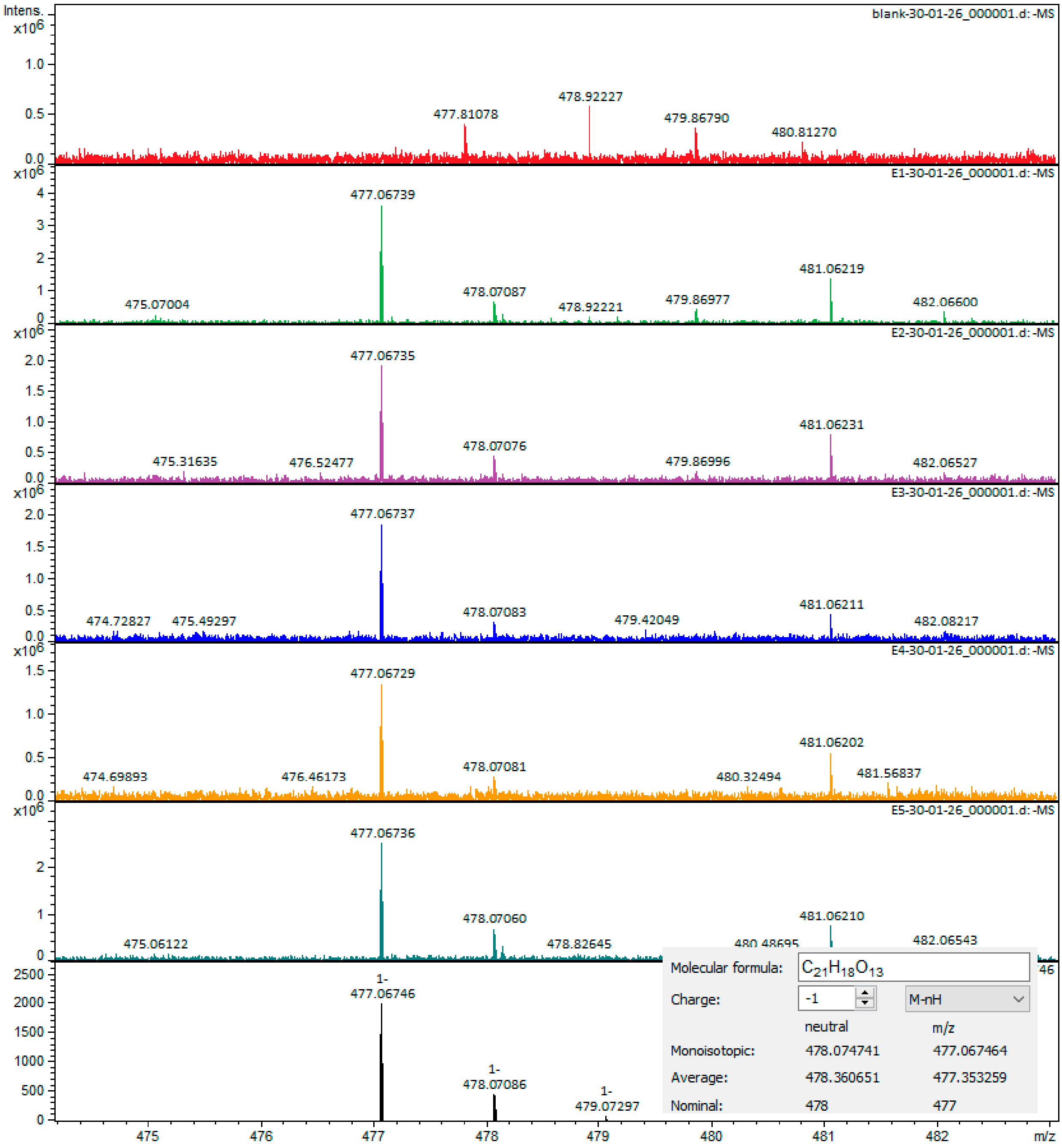

Figure S13. FT-ICR MS spectrum of a quercetin derivative ($C_{21}H_{18}O_{13}$) detected in eucalyptus extract (E1-E5) in negative ionization mode (ESI-). The deprotonated molecular ion $[M+H]^-$ at m/z ~477 is highlighted.

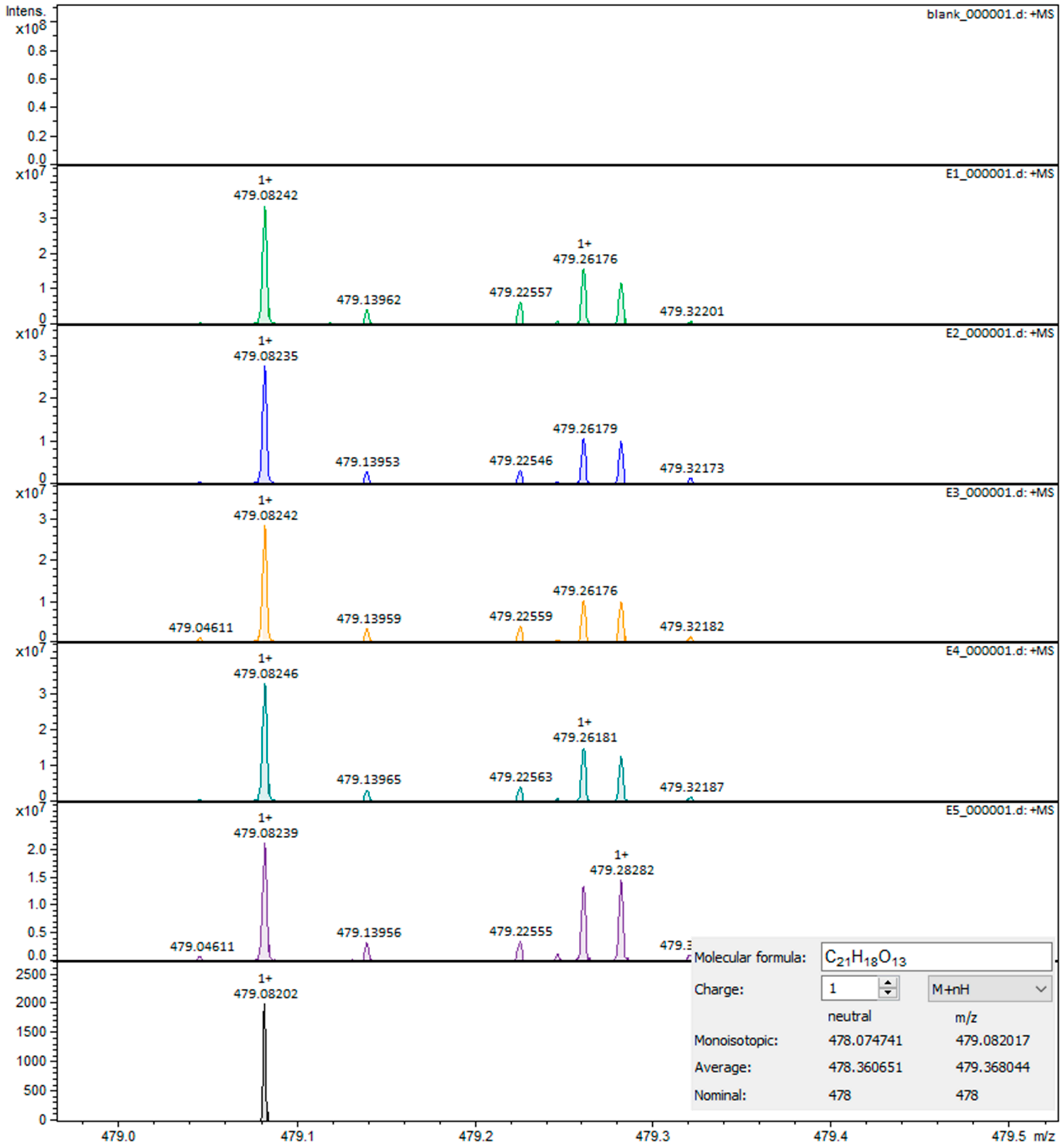

Figure S14. FT-ICR MS spectrum of a quercetin derivative ($C_{21}H_{18}O_{13}$) detected in eucalyptus extract (E1-E5) in positive ionization mode (ESI+). The protonated molecular ion $[M+H]^+$ at m/z ~479 is highlighted.

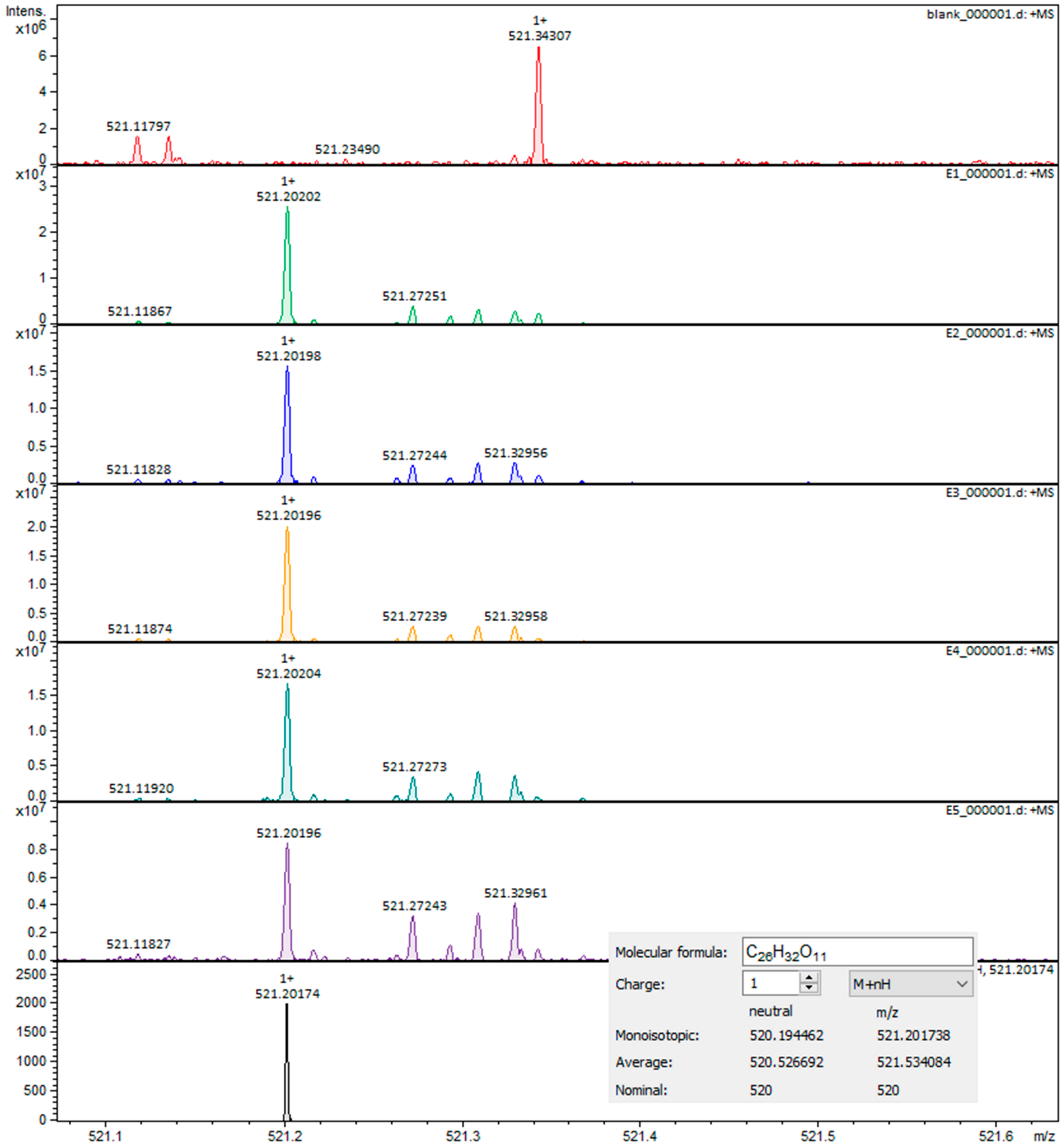

Figure S15. FT-ICR MS spectrum of quercitrin ($C_{21}H_{20}O_{11}$) detected in eucalyptus extract (E1-E5) in positive ionization mode (ESI+). The protonated molecular ion $[M+H]^+$ at m/z ~521 is highlighted.

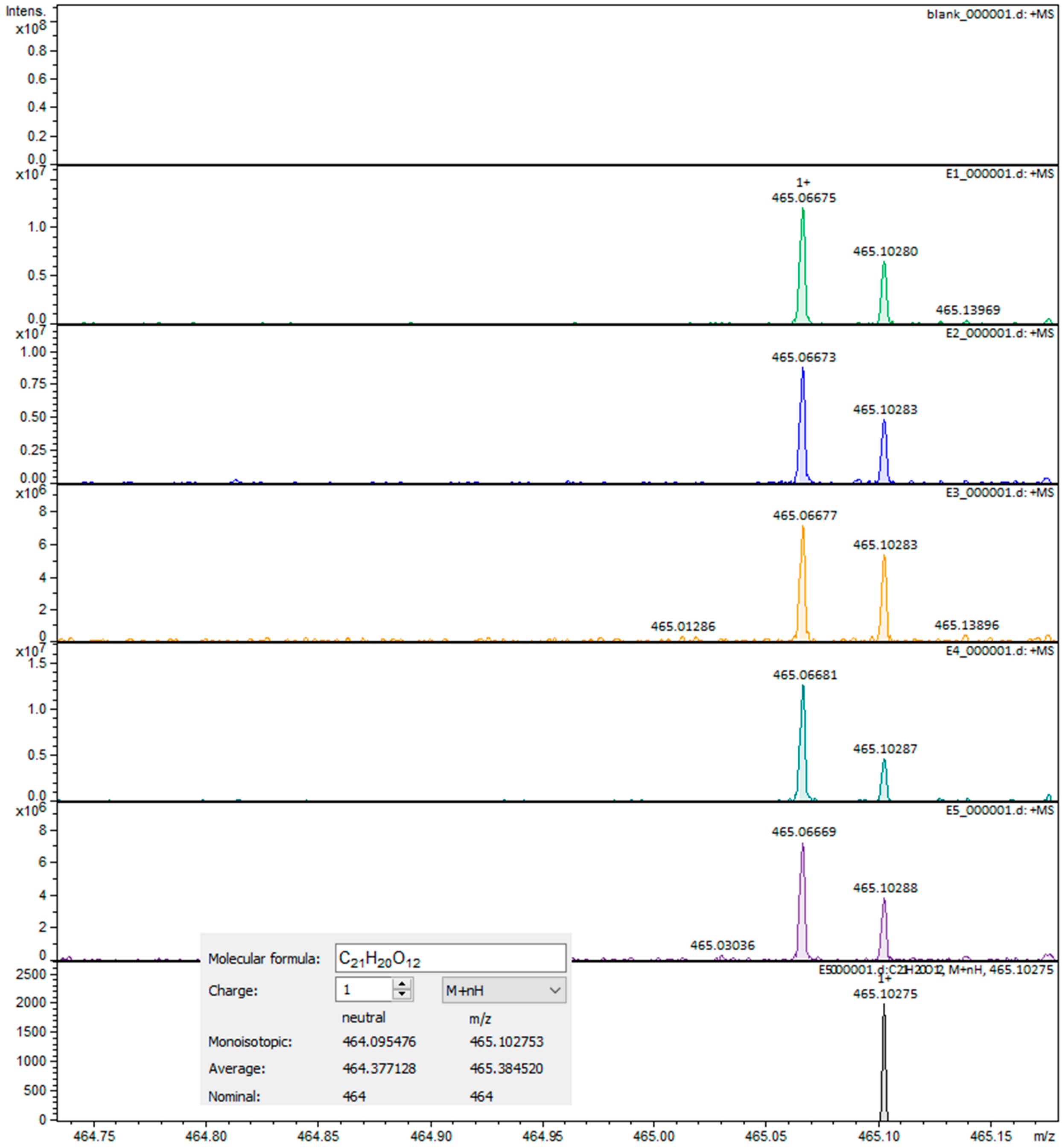

Figure S16. FT-ICR MS spectrum of isoquercitrin ($C_{21}H_{20}O_{12}$) detected in eucalyptus extract (E1-E5) in positive ionization mode (ESI+). The protonated molecular ion $[M+H]^+$ at m/z ~465 is highlighted.

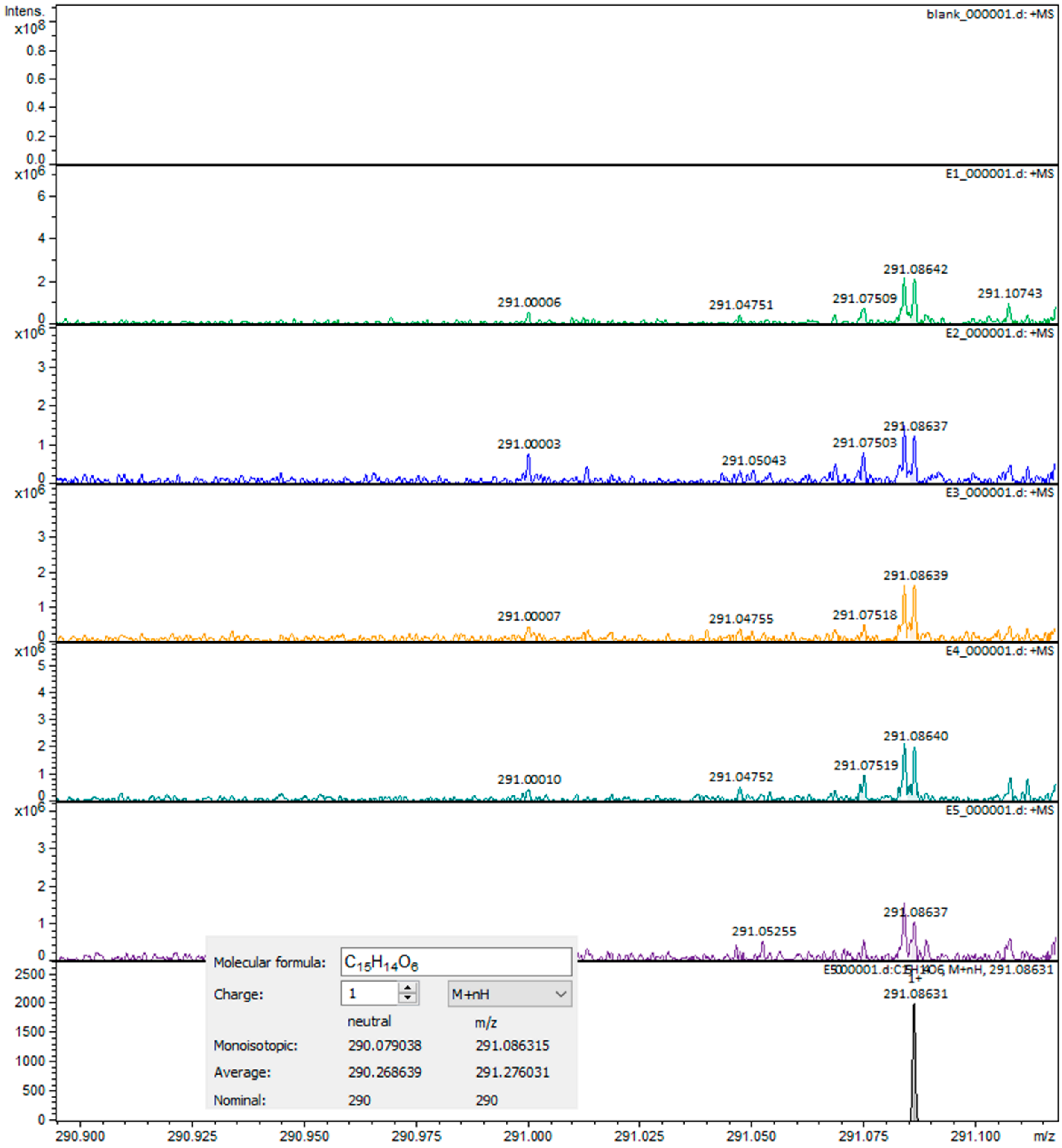

Figure S17. FT-ICR MS spectrum o catechin/epicatechin flavonoid ($C_{15}H_{14}O_6$) detected in eucalyptus extract (E1-E5) in positive ionization mode (ESI+). The protonated molecular ion $[M+H]^+$ at m/z ~291 is highlighted.

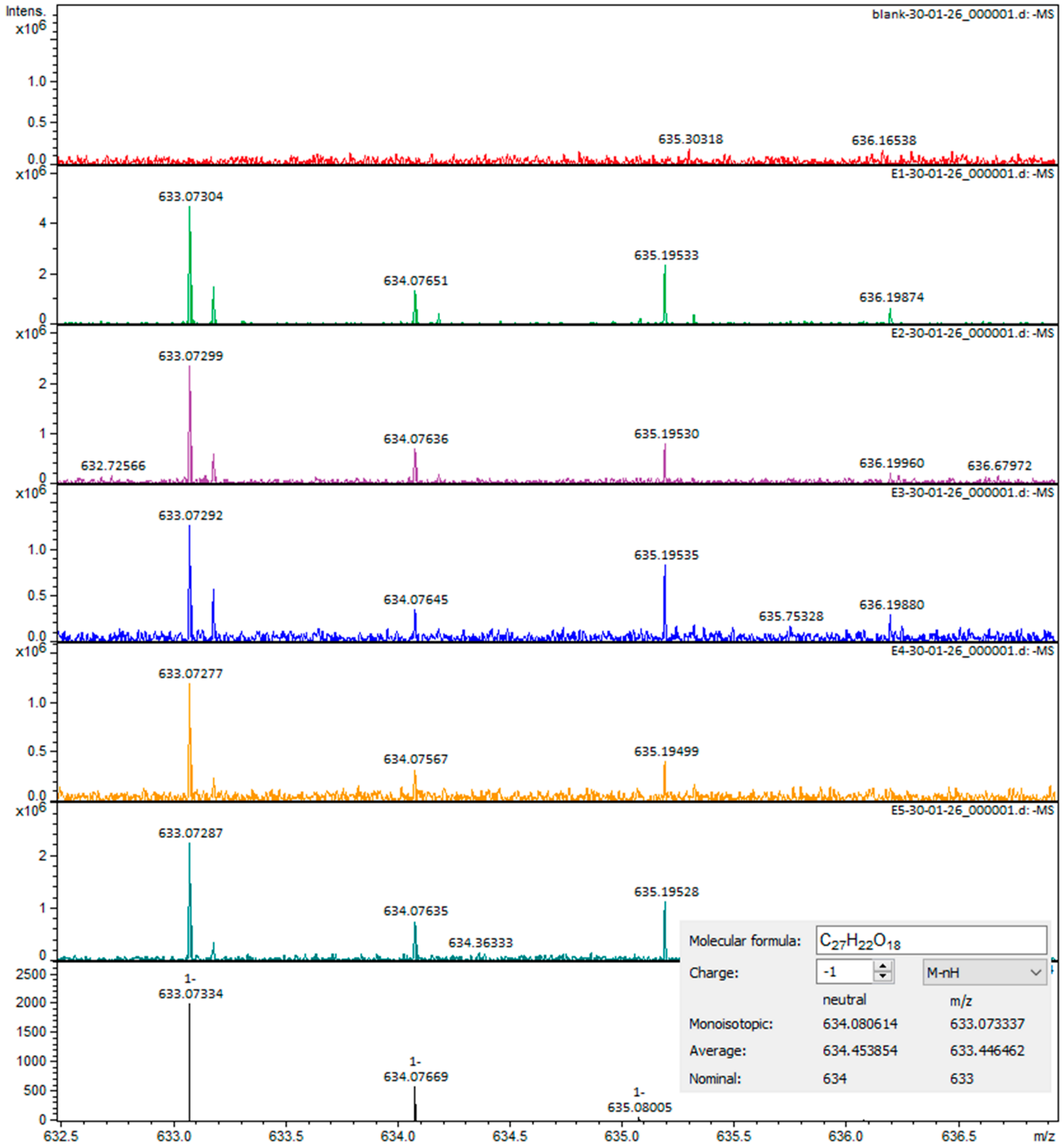

Figure S18. FT-ICR MS spectrum of corilagin ($C_{27}H_{22}O_{18}$) detected in eucalyptus extract (E1-E5) in negative ionization mode (ESI-). The deprotonated molecular ion $[M+H]^-$ at m/z ~633 is highlighted.

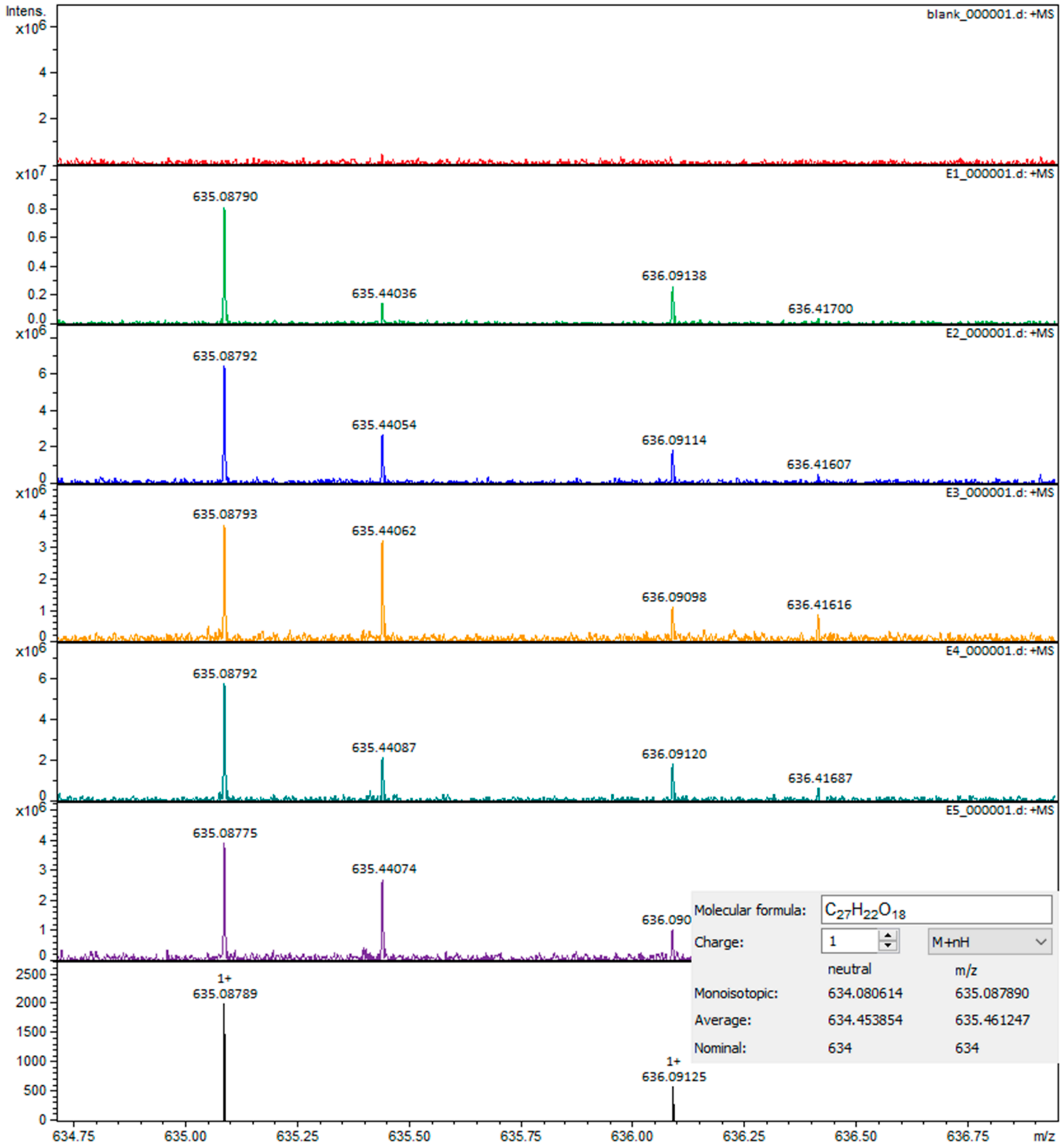

Figure S19. FT-ICR MS spectrum of corilagin ($C_{27}H_{22}O_{18}$) detected in eucalyptus extract (E1-E5) in positive ionization mode (ESI+). The protonated molecular ion $[M+H]^+$ at m/z ~635 is highlighted.

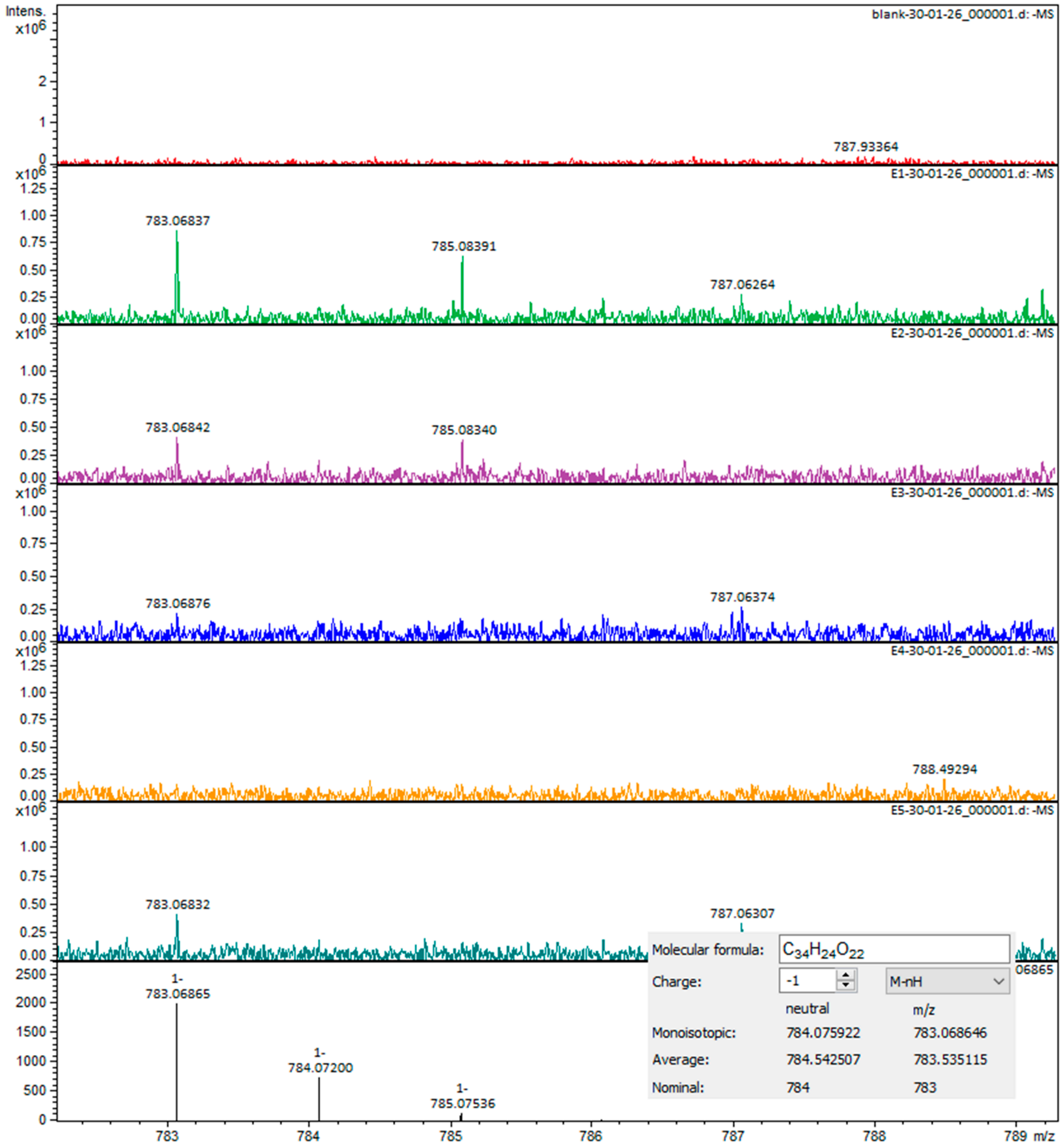

Figure S20. FT-ICR MS spectrum of pedunculagin ($C_{34}H_{24}O_{22}$) detected in eucalyptus extract (E1-E5) in negative ionization mode (ESI-). The deprotonated molecular ion $[M+H]^-$ at m/z ~783 is highlighted.

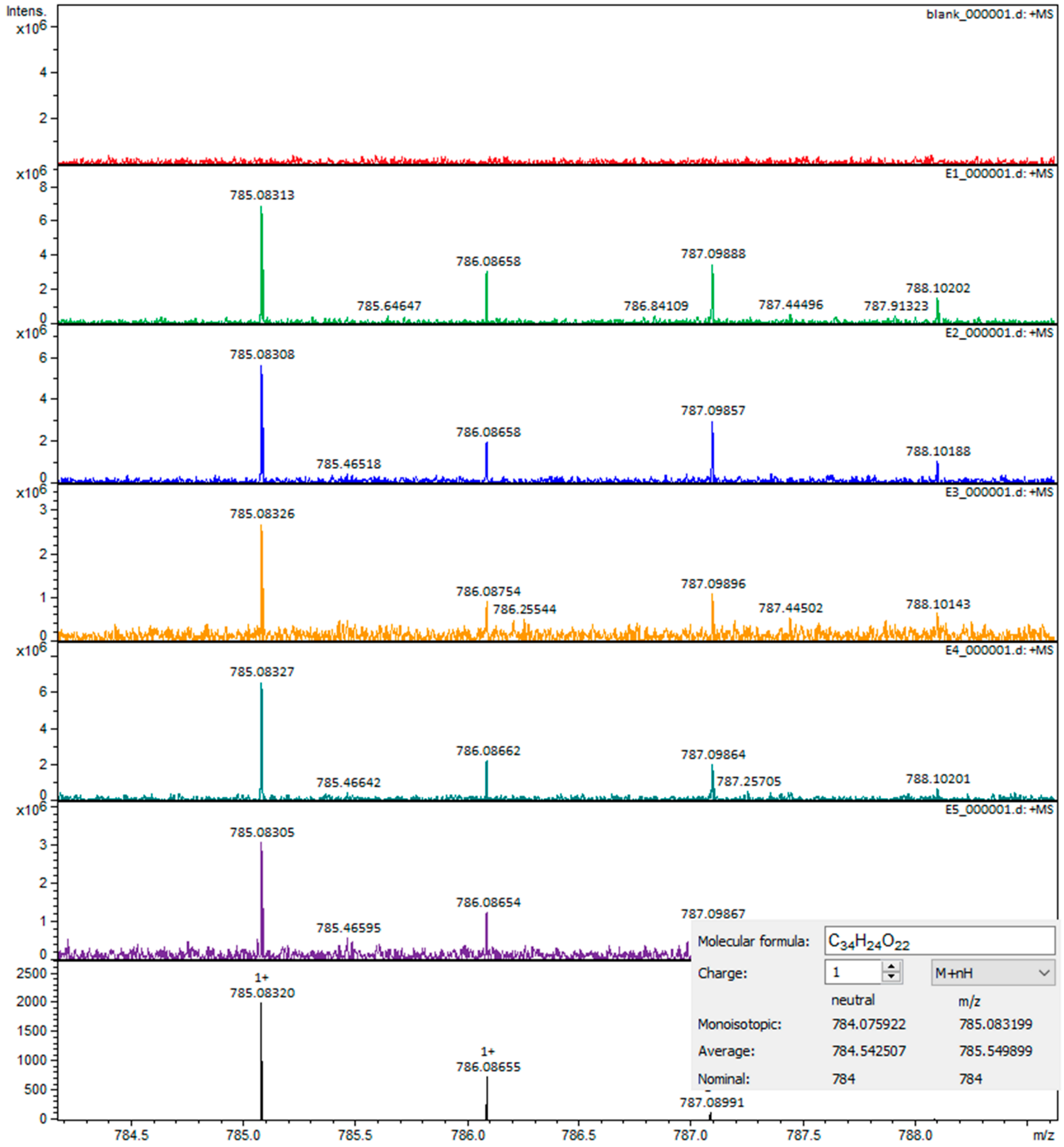

Figure S21. FT-ICR MS spectrum of pedunculagin ($C_{34}H_{24}O_{22}$) detected in eucalyptus extract (E1-E5) in positive ionization mode (ESI+). The protonated molecular ion $[M+H]^+$ at m/z ~785 is highlighted.

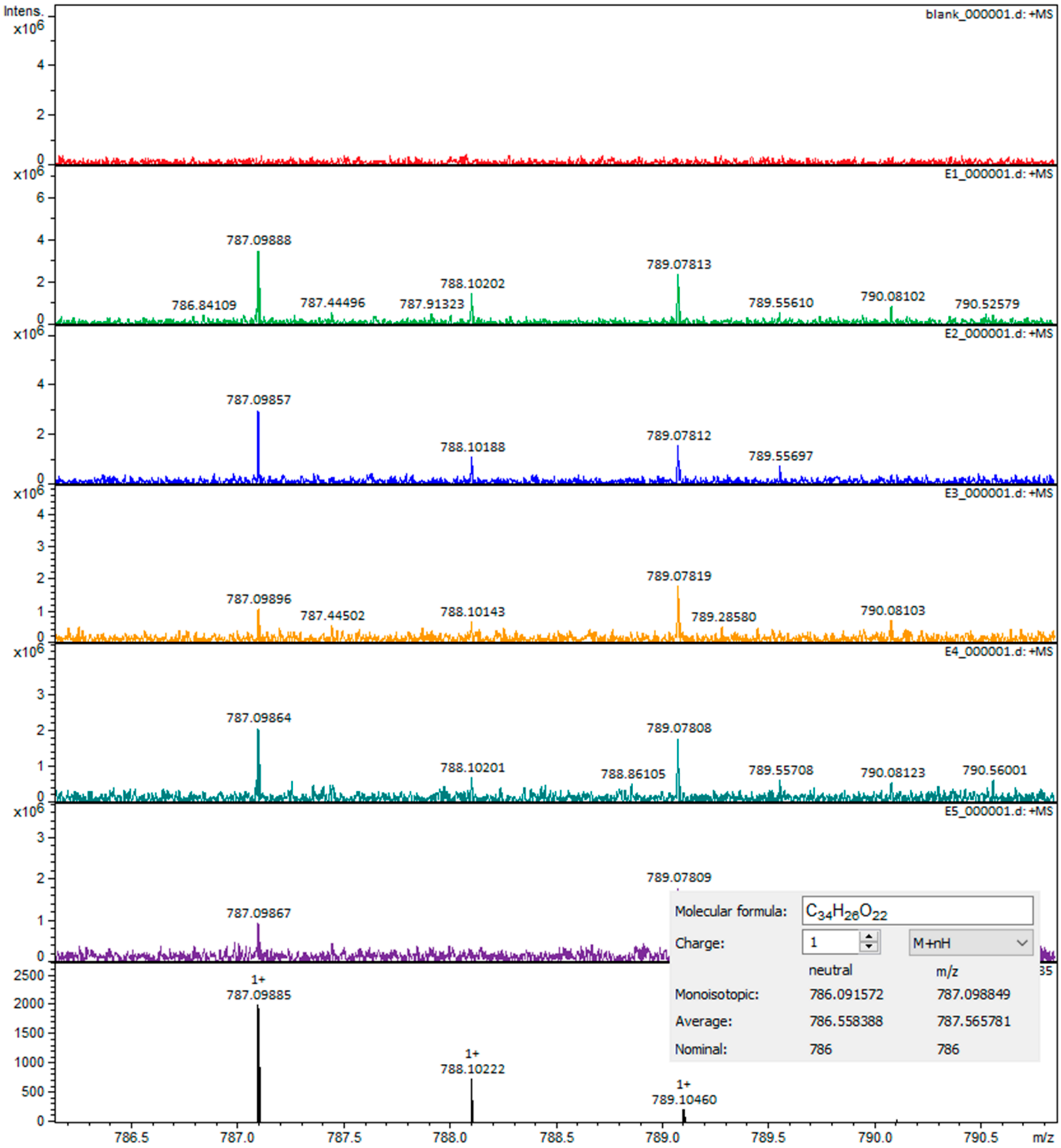

Figure S22. FT-ICR MS spectrum of tellimagrandin I ($C_{34}H_{26}O_{22}$) detected in eucalyptus extract (E1-E5) in positive ionization mode (ESI+). The protonated molecular ion $[M+H]^+$ at m/z ~787 is highlighted.

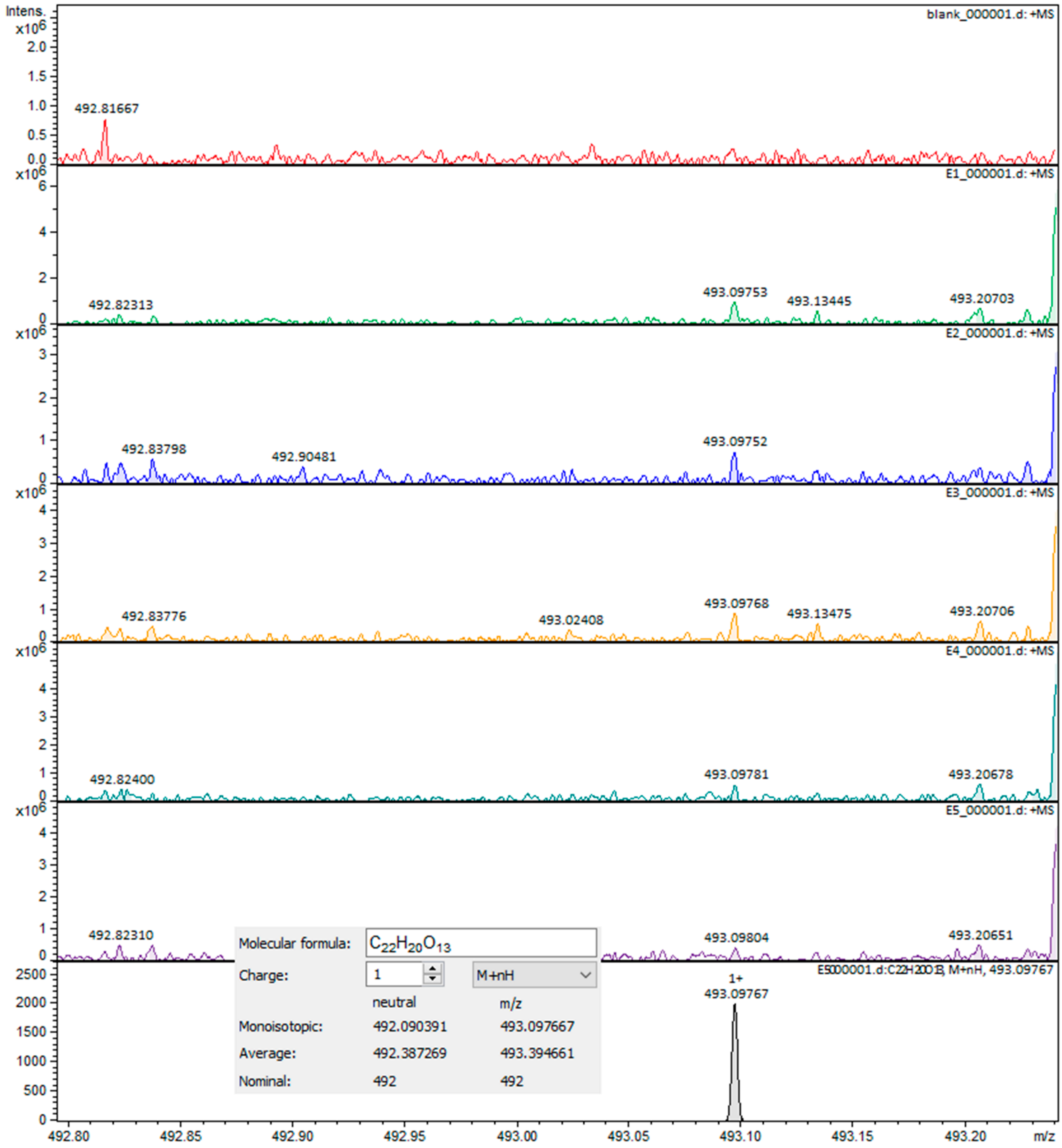

Figure S23. FT-ICR MS spectrum of a dimethyl ellagic acid glycoside ($C_{22}H_{20}O_{13}$) detected in eucalyptus extract (E1-E5) in positive ionization mode (ESI+). The protonated molecular ion $[M+H]^+$ at m/z ~493 is highlighted.

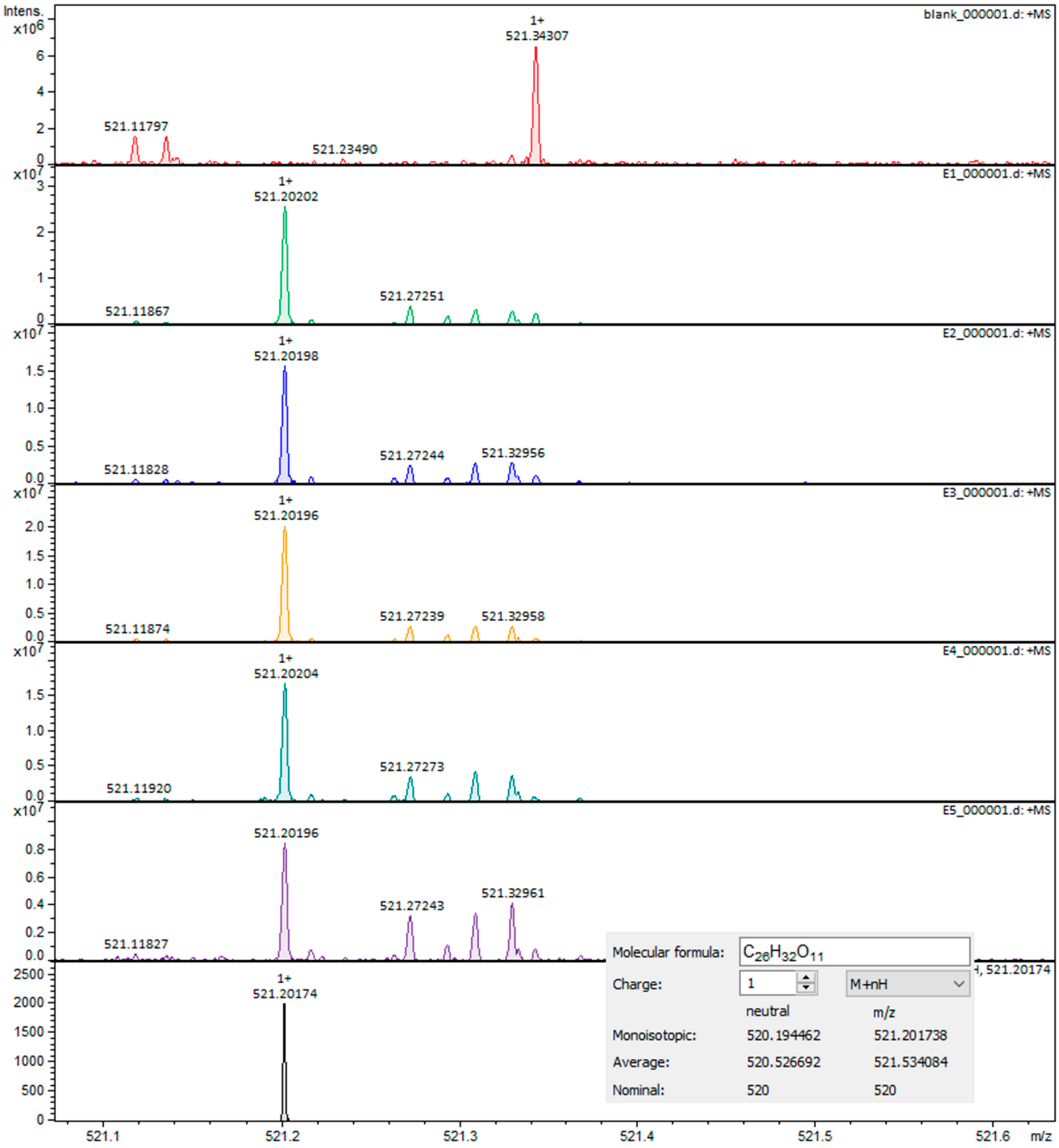

Figure S24. FT-ICR MS spectrum of cypellocarpin C ($C_{26}H_{32}O_{11}$) detected in eucalyptus extract (E1-E5) in positive ionization mode (ESI+). The protonated molecular ion $[M+H]^+$ at m/z ~521 is highlighted.

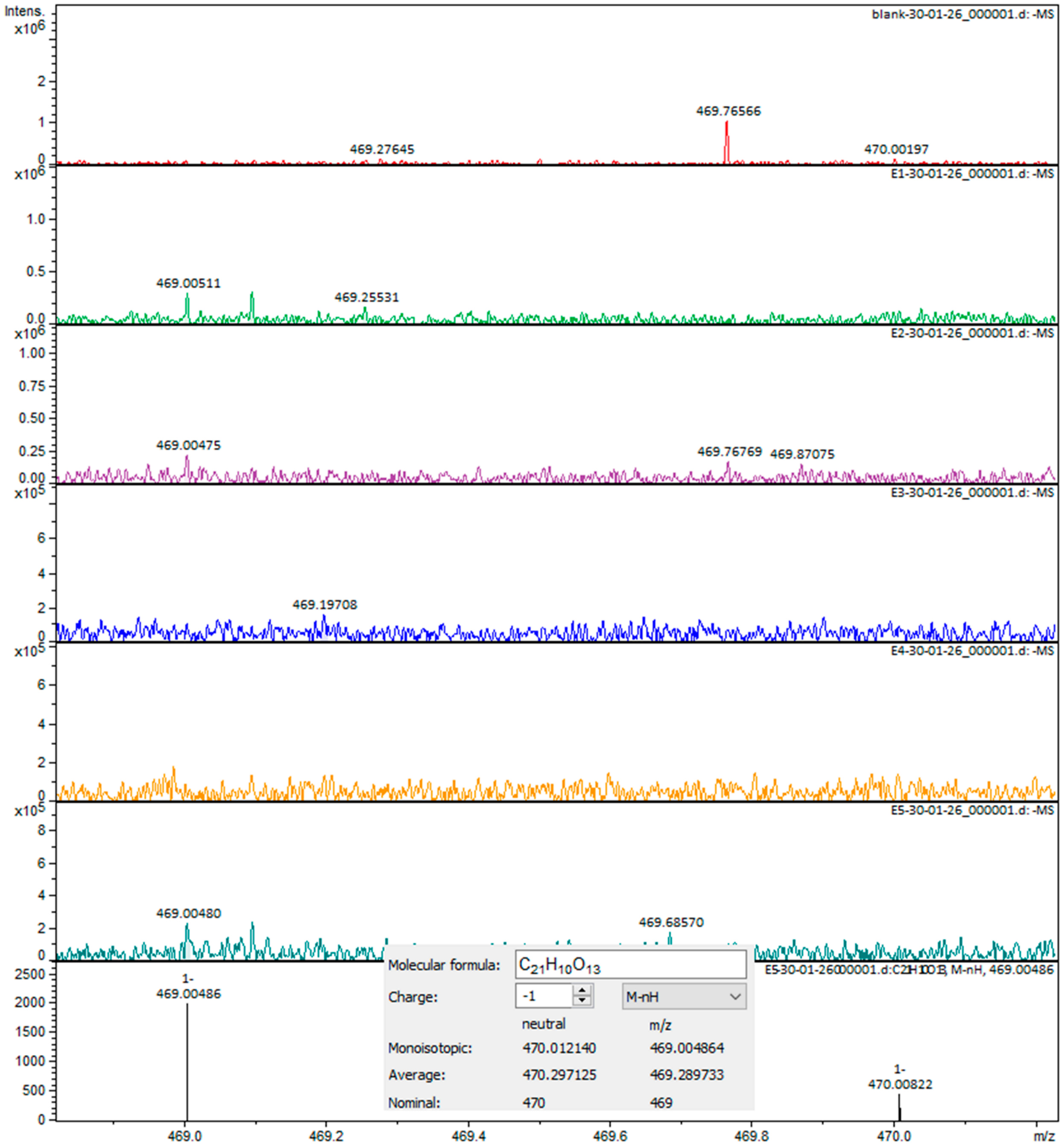

Figure S25. FT-ICR MS spectrum of a hydrolysable tannins-related molecule ($C_{21}H_{10}O_{13}$) detected in eucalyptus extract (E1-E5) in negative ionization mode (ESI-). The deprotonated molecular ion $[M+H]^-$ at m/z ~469 is highlighted.

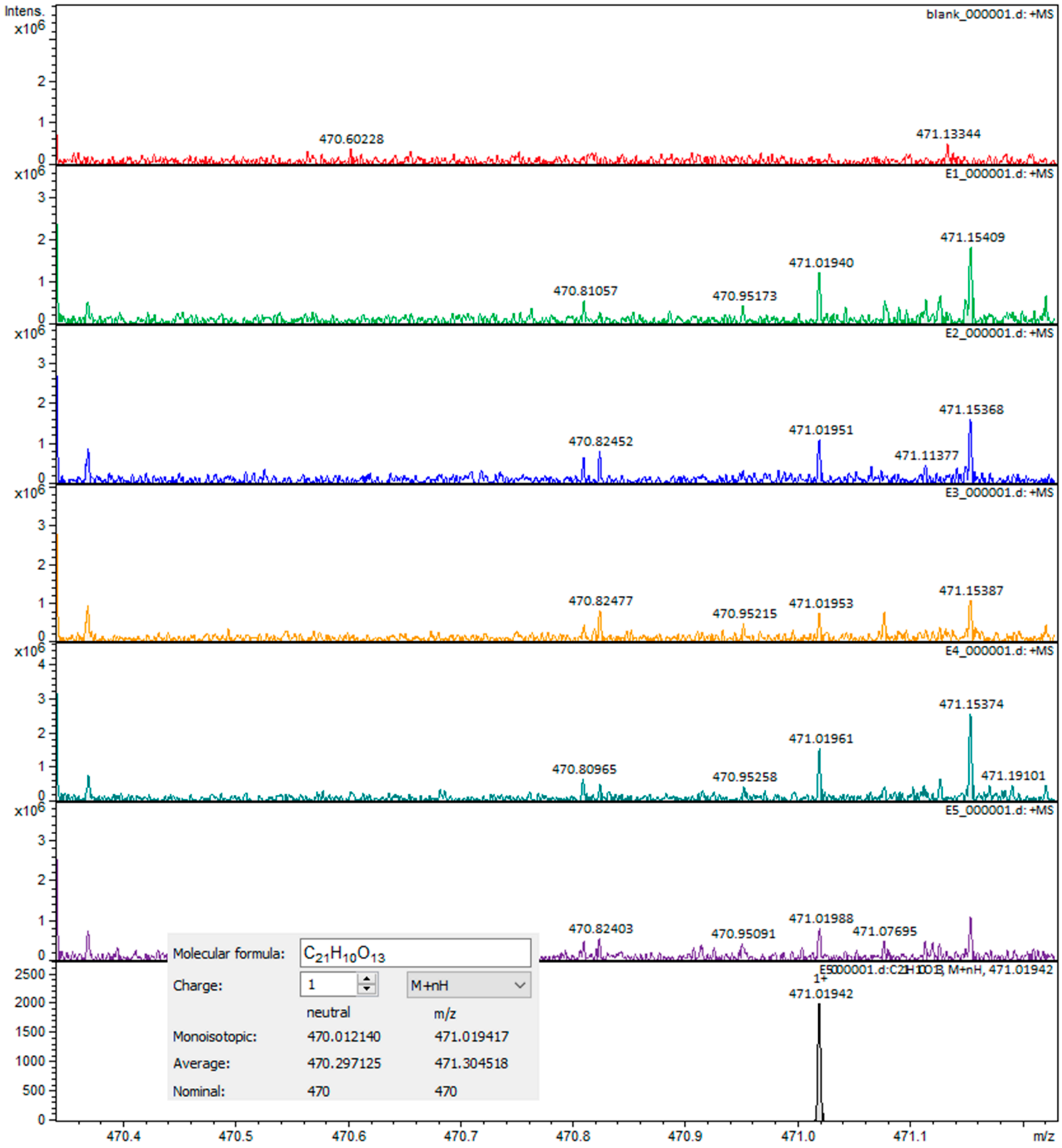

Figure S26. FT-ICR MS spectrum of a hydrolysable tannins-related molecule ($C_{21}H_{10}O_{13}$) detected in eucalyptus extract (E1-E5) in positive ionization mode (ESI+). The protonated molecular ion $[M+H]^+$ at m/z ~471 is highlighted.

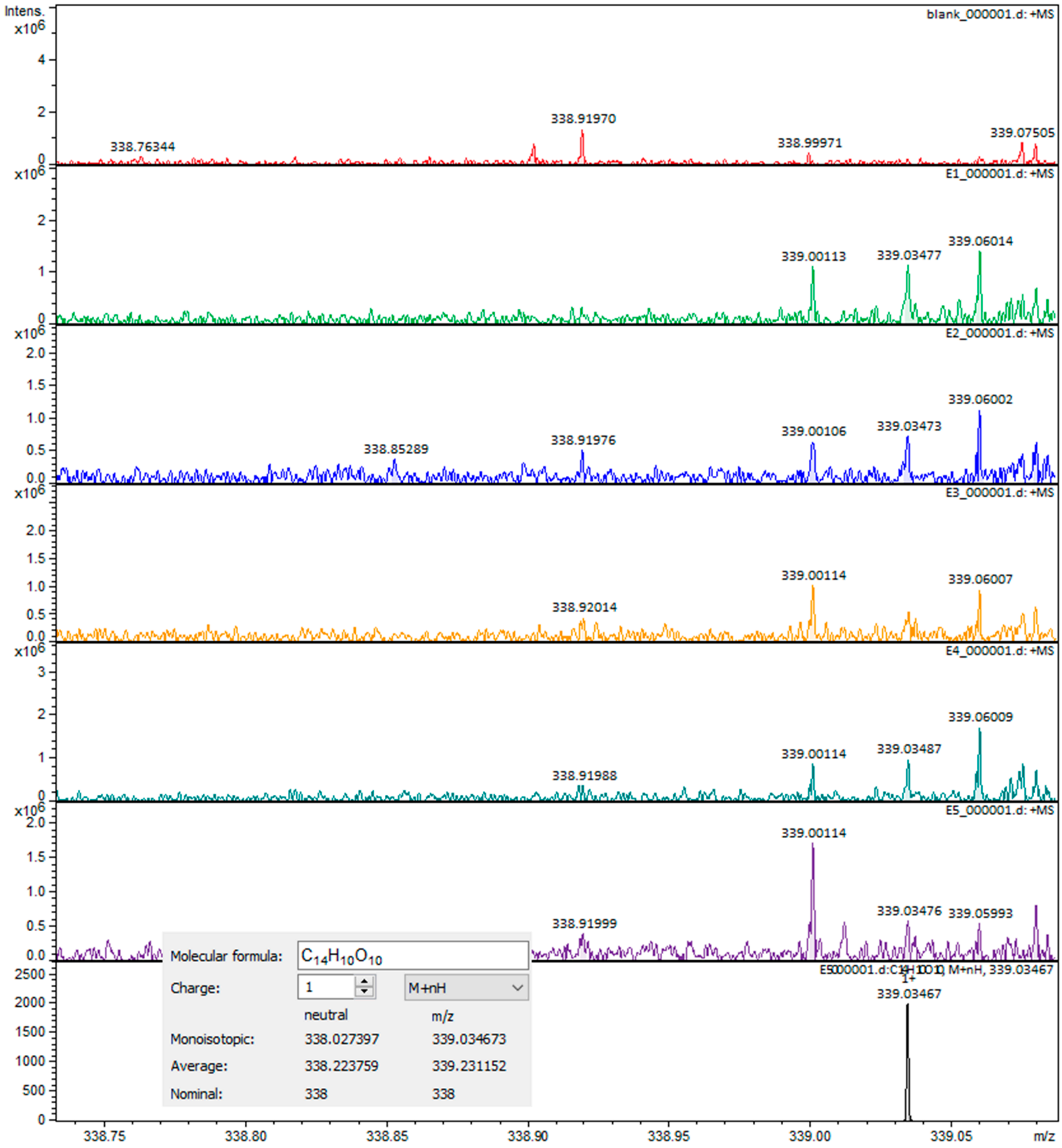

Figure S27. FT-ICR MS spectrum of a hydrolysable tannins-related molecule ($C_{14}H_{10}O_{10}$) detected in eucalyptus extract (E1-E5) in positive ionization mode (ESI+). The protonated molecular ion $[M+H]^+$ at m/z ~339 is highlighted.

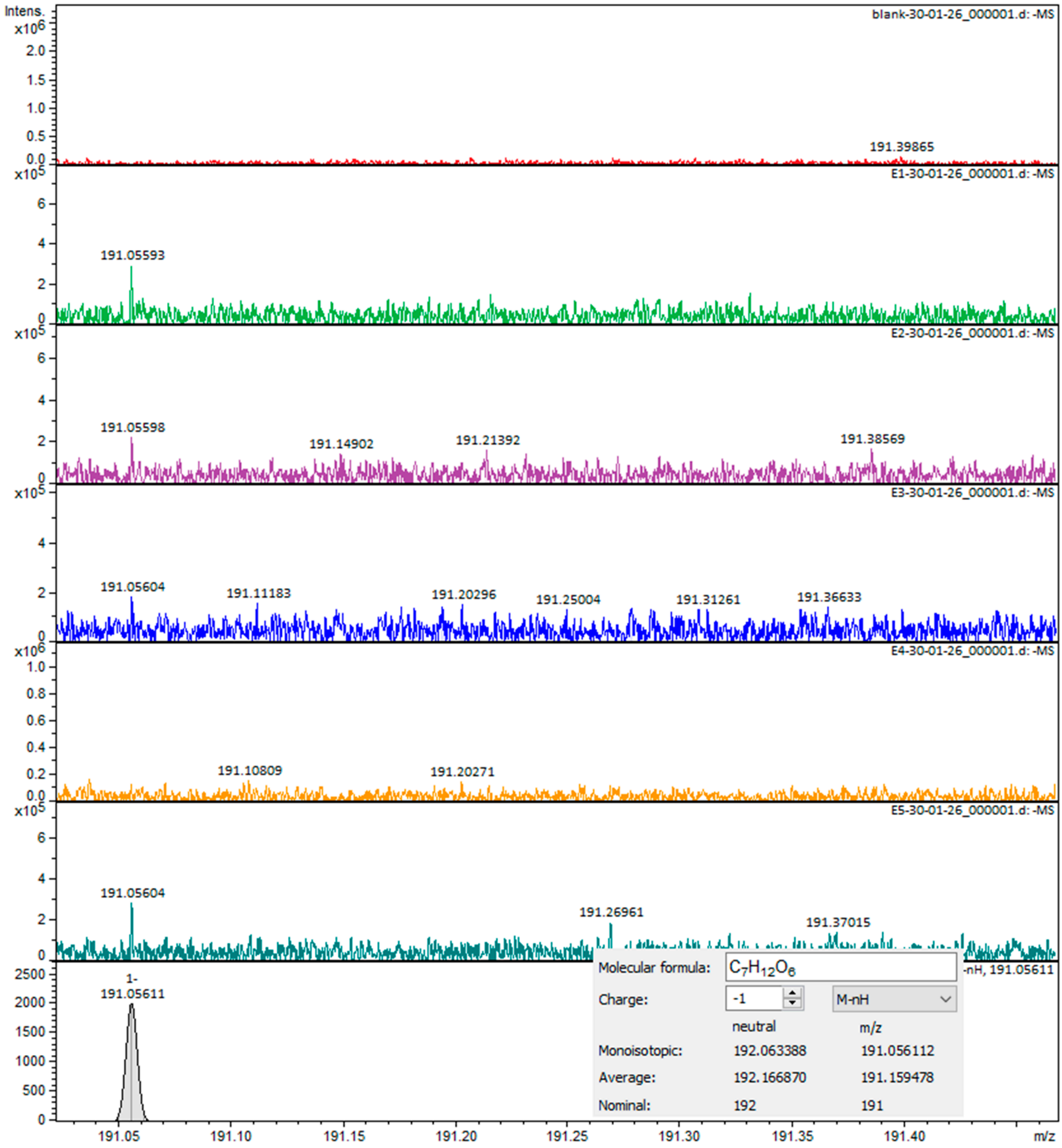

Figure S28. FT-ICR MS spectrum of quinic acid ($C_7H_{12}O_6$) detected in eucalyptus extract (E1-E5) in negative ionization mode (ESI-). The deprotonated molecular ion $[M+H]^-$ at m/z ~191 is highlighted.

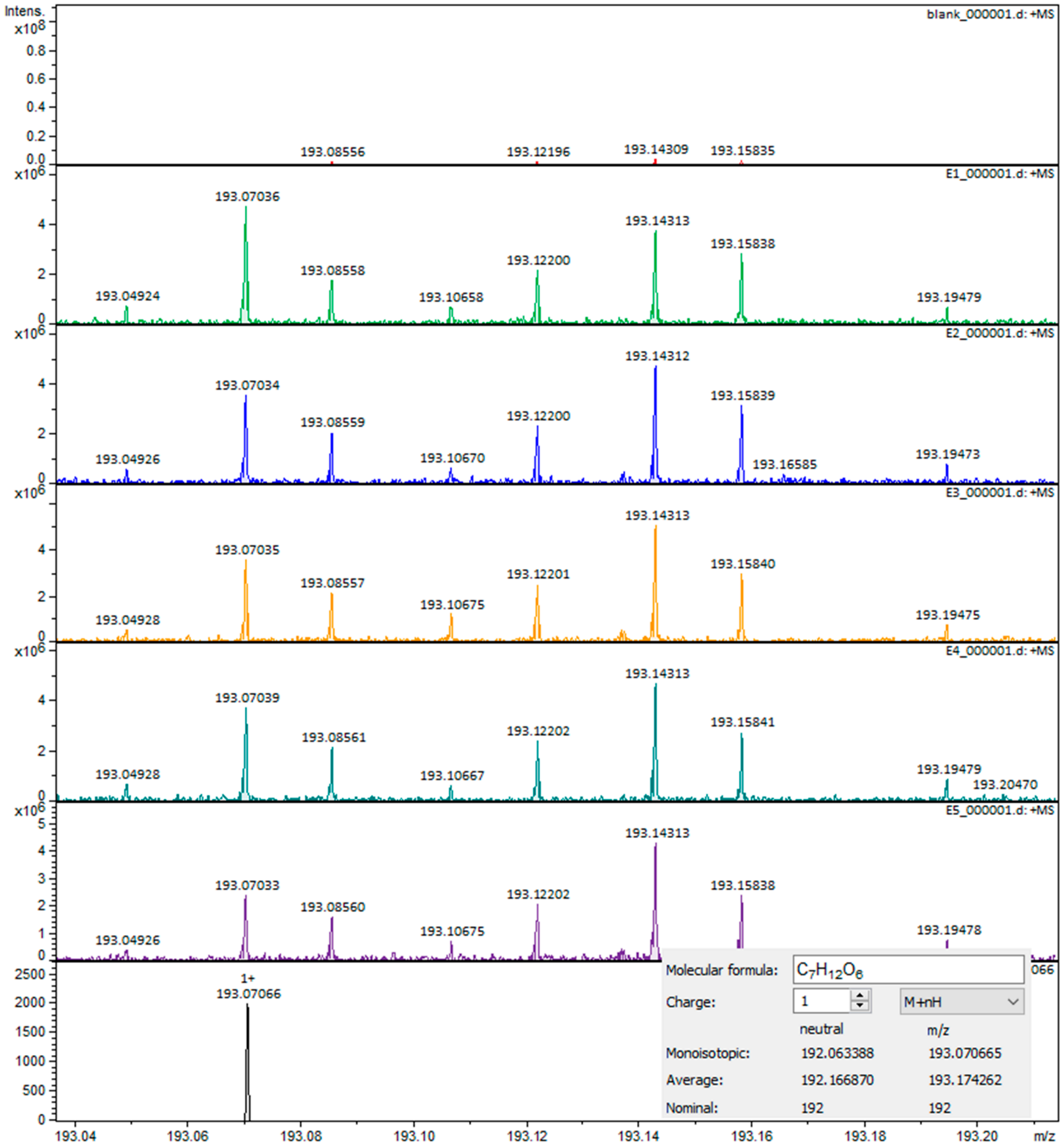

Figure S29. FT-ICR MS spectrum of quinic acid ($C_7H_{12}O_6$) detected in eucalyptus extract (E1-E5) in positive ionization mode (ESI+). The protonated molecular ion $[M+H]^+$ at m/z ~193 is highlighted.

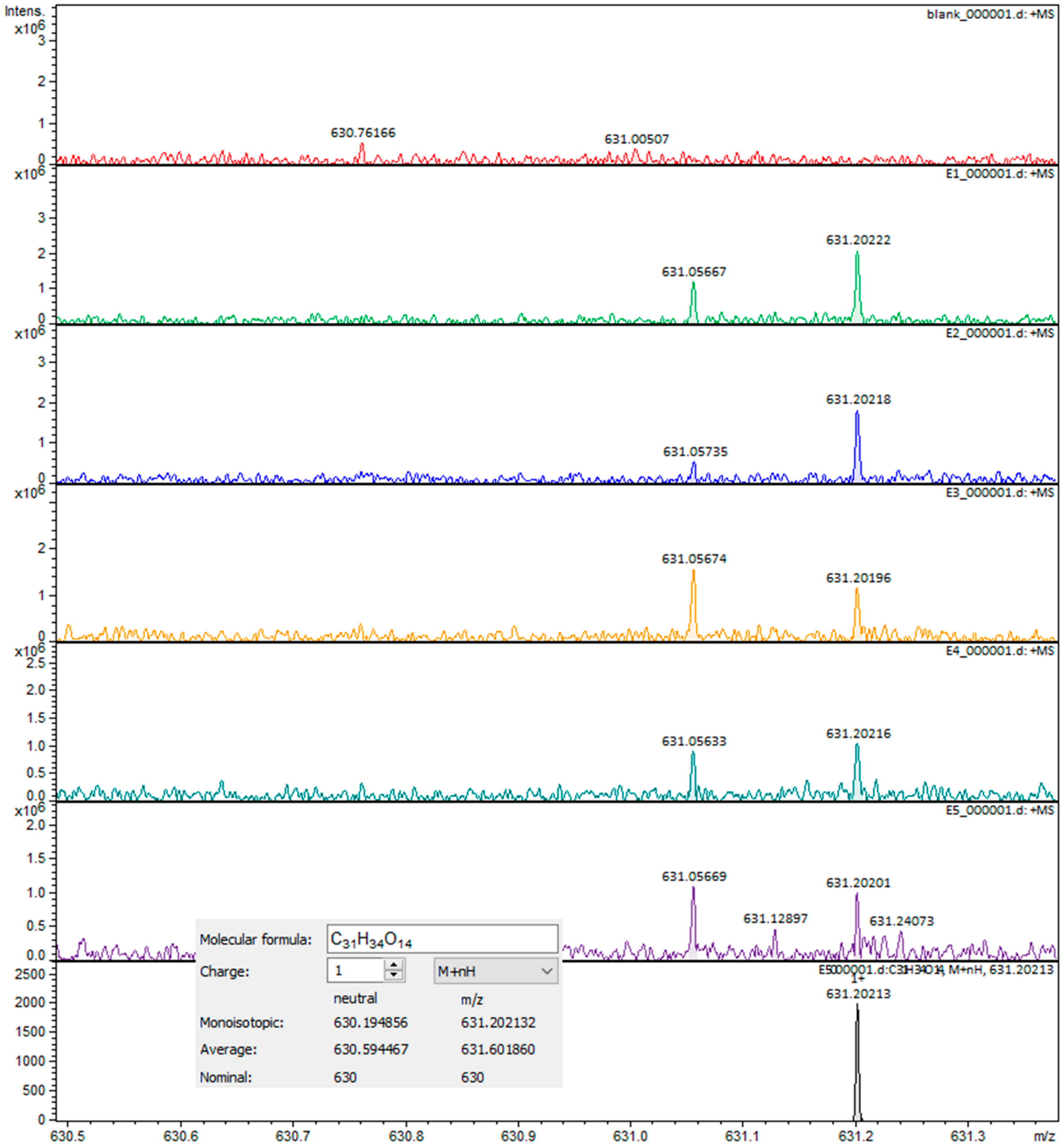

Figure S30. FT-ICR MS spectrum of unassigned compound ($C_{31}H_{34}O_{14}$) detected in eucalyptus extract (E1-E5) in positive ionization mode (ESI+). The protonated molecular ion $[M+H]^+$ at m/z ~631 is highlighted.

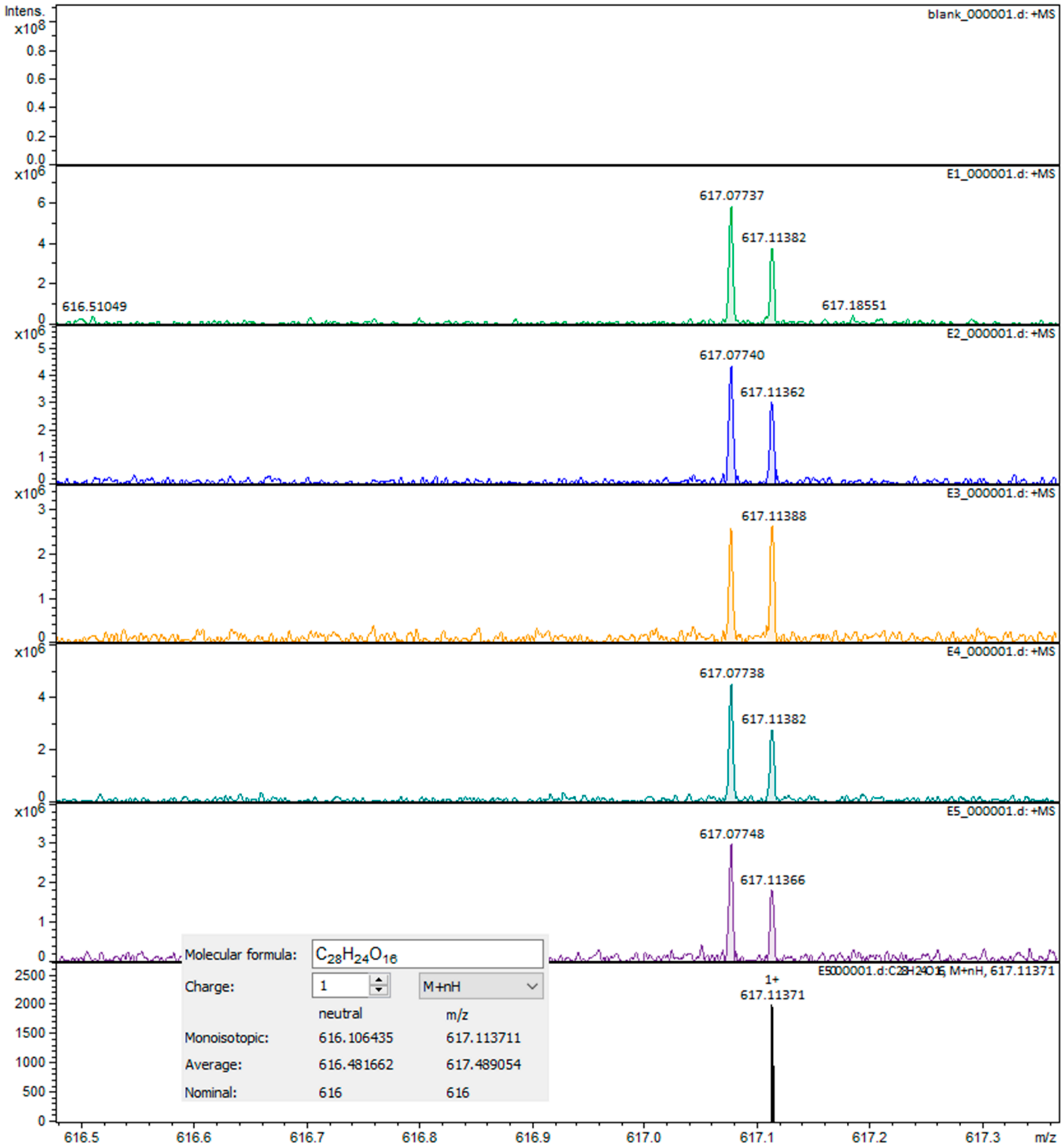

Figure S31. FT-ICR MS spectrum of unassigned compound ($C_{28}H_{24}O_{16}$) detected in eucalyptus extract (E1-E5) in positive ionization mode (ESI+). The protonated molecular ion $[M+H]^+$ at m/z ~617 is highlighted.

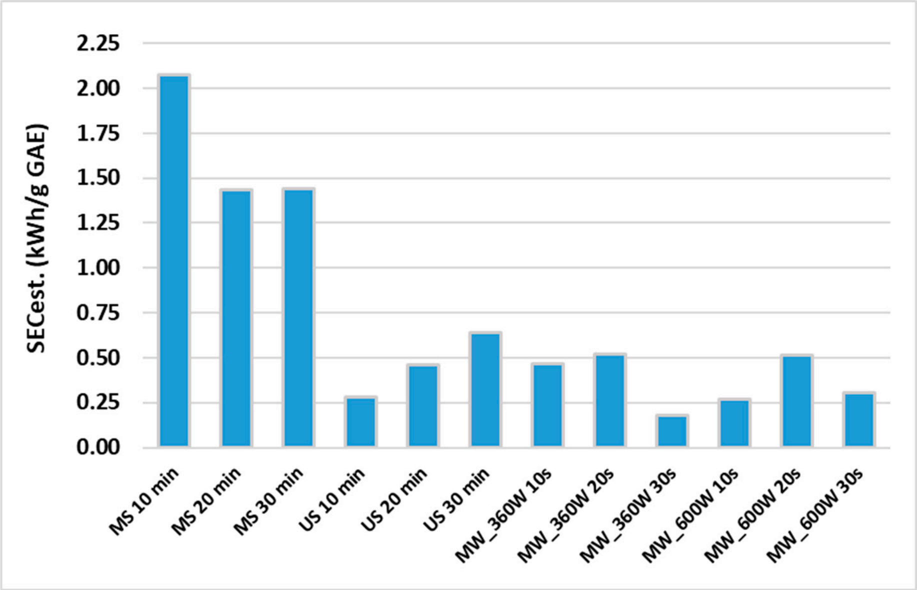

Figure S32. Estimated specific energy demand related to phenolic species recovery from *Eucalyptus globulus* leaves
